# Emerging RNA-Based Therapeutic and Diagnostic Options: Recent Advances and Future Challenges in Genitourinary Cancers

**DOI:** 10.3390/ijms24054601

**Published:** 2023-02-27

**Authors:** Fabiana Tortora, Evelina La Civita, Pankaj Trivedi, Ferdinando Febbraio, Daniela Terracciano, Amelia Cimmino

**Affiliations:** 1 Institute of Genetics and Biophysics “A. Buzzati Traverso”, National Research Council (CNR), 80131 Naples, Italy; 2 Department of Translational Medical Sciences, University of Naples “Federico II”, 80131 Naples, Italy; 3 Department of Experimental Medicine, La Sapienza University, Viale Regina Elena 324, 00161 Rome, Italy; 4 Institute of Biochemistry and Cell Biology, National Research Council (CNR), 80131 Naples, Italy

**Keywords:** genitourinary cancer, renal cell carcinoma, bladder cancer, prostate cancer, long non-coding RNA, molecular biomarkers

## Abstract

Renal cell carcinoma, bladder cancer, and prostate cancer are the most widespread genitourinary tumors. Their treatment and diagnosis have significantly evolved over recent years, due to an increasing understanding of oncogenic factors and the molecular mechanisms involved. Using sophisticated genome sequencing technologies, the non-coding RNAs, such as microRNAs, long non-coding RNAs, and circular RNAs, have all been implicated in the occurrence and progression of genitourinary cancers. Interestingly, DNA, protein, and RNA interactions with lncRNAs and other biological macromolecules drive some of these cancer phenotypes. Studies on the molecular mechanisms of lncRNAs have identified new functional markers that could be potentially useful as biomarkers for effective diagnosis and/or as targets for therapeutic intervention. This review focuses on the mechanisms underlying abnormal lncRNA expression in genitourinary tumors and discusses their role in diagnostics, prognosis, and treatment.

## 1. Introduction

The treatment of genitourinary cancers has received considerable attention in recent years. These tumors comprise a heterogeneous group that includes renal cell carcinoma (RCC), bladder cancer (BlCa), and prostate cancer (PCa) [1]. Different research strategies have been implemented to identify novel biomarkers for these tumors [2]. In facilitating an early and accurate diagnosis, biomarkers can be quite useful in improving clinical disease management and reducing the use of invasive diagnostic methods [1,2]. Furthermore, clinicians may be able to lessen side effects and cost by choosing the best treatment plan for patients exhibiting certain biomarkers [1]. In particular, the identification of specific targets/biomarkers has led to the most recent significant advancements in the development of immunotherapies, especially of immune checkpoint inhibitors or immunomodulatory agents of the tumor microenvironment [2]. Overall survival has risen with immunotherapy and this therapeutic approach also has reduced the onset of metastases in some patients and revolutionized management of malignant tumors in the genitourinary system [2].

Among the currently available immune checkpoints, the following two have often been targeted with corresponding antibodies: (1) PD-1, a receptor found on the surface of T lymphocytes that have been activated, as well as its ligand, PD-L1, which is expressed on antigen presenting/tumor cells, modulates T-cell activity, and inhibits the antitumor immune response; and (2) CTLA-4, which suppresses the immune response and is expressed by activated T cells [2]. For the treatment of urothelial carcinoma and RCC, which are classified as immunologically “hot” malignancies, a number of checkpoint inhibitors (CPI) have been approved [3]. RCCs have higher neoantigen loads and higher levels of tumor-infiltrating lymphocytes (TILs), which make these neoplasms more readily identifiable by the immune system and prompt an intense immunological response [3].

The intricacy of the tumor microenvironment influences T-cell activation against malignancies. However, it is generally known that only a small percentage of patients, even those with immunologically “hot” malignancies, respond to CPIs [3].

The varying reactions to various medicines can be partially explained by the molecular heterogeneity of malignancies. As part of the immunotherapeutic approach, new drugs are continuously being sought after which are specifically suited to a range of targets, such as tyrosine kinase inhibitors, mTOR inhibitors, and new fusion proteins [4]. In this scenario, we also find the most recent data on non-coding RNAs. In particular, long non-coding RNAs (lncRNAs), transcribed throughout the human genome, have been revealed as novel markers using advanced genome sequencing technologies. LncRNAs, a class of RNA molecules with a length of more than 200 nucleotides, are crucial for the emergence and growth of malignancies. In particular, regulation of cell migration, invasion, the cell cycle, epithelial-mesenchymal transition (EMT), DNA damage, and drug resistance are greatly influenced by lncRNAs [4].

Drug discovery is advancing quickly right now and there are several ongoing preclinical investigations and promising clinical trials. This review focuses on the use of lncRNAs as biomarkers for cancer diagnosis and/or prognosis, as well as development of novel therapeutics, which are predicted to continue to improve outcomes in patients with genitourinary malignancies.

## 2. Renal Cell Carcinoma

RCC is the most widespread kidney malignancy in adults, accounting for 3.7% of all tumors in the world. This tumor is also the main cause of cancer-related morbidity and mortality worldwide [5]. The most prevalent subtype of RCC is clear cell renal cell carcinoma (ccRCC), and comprehending the molecular changes associated with malignant transformation is crucial to achieving longer survival [6].

Currently, RCC treatment and diagnostic methods are few and mostly restricted to advanced disease stages. There is an increasing need to include new prognostic and predictive biomarkers, which can also serve as therapeutic targets, because practically all subtypes of RCC are resistant to chemotherapy and radiotherapy [7].

The connection between lncRNAs and RCC has recently received attention, and certain significant lncRNA molecules have been identified as biological markers and therapeutic targets. In particular, lncRNAs contribute to cancer phenotypes by interacting with proteins, DNA, and RNA [7]. Recent research on the molecular mechanisms of lncRNAs has clarified their function in the development of malignant tumors, making them suitable targets for cancer treatment and detection. Several cancer phenotypes are the result of dysfunctions in intrinsic cellular regulatory networks and intercellular communications that are conducive to the tumor microenvironment [8,9]. An interesting example is the association between increased MALAT1 expression in the early diagnosis of lymph node metastases and poor survival in RCC patients [10,11]. In ccRCC, MALAT1 is overexpressed and plays an important role in regulating epithelial–mesenchymal transition (EMT) via its well-documented interaction with EZH2 [12]. Furthermore, miRNAs, the small, single-stranded, 18–25-nucleotides-long cellular RNAs that do not code for proteins and are evolutionarily highly conserved, play a relevant role in carcinogenesis. They contribute to cancer development by controlling cell growth, proliferation, angiogenesis, invasion, and migration and function as either oncogenic or suppressor miRs [13]. Thus, the differential expression of miRNAs can serve as a key marker for the diagnosis and prognosis of tumors as well as a possible therapeutic target. It has been demonstrated that lncRNAs function as “molecular sponges” for miRNAs to prevent their “silencing impact” on target miRNAs [13].

MALAT1 has also been demonstrated to promote EMT in ccRCC cells by functioning as a competing endogenous RNA (ceRNA) and preventing miRNA-mediated degradation of the transcript-encoding zinc finger E box binding homeobox 2 (ZEB2), a transcriptional regulator of E-cadherin [14]. Regarding the mechanisms that control transcription, it was found that the HIF pathway is required for transcription factor FOS to upregulate MALAT1 in ccRCC [12]. Additional research revealed that shorter survival and bad clinicopathological characteristics were linked to increased lncRNA ZFAS1 expression. Furthermore, ZFAS1 knockdown inhibited migration, invasion, and proliferation of ccRCC cells [15]. 

Compared to nearby non-tumor tissues, it was discovered that the expression of lncRNA ZFAS1 in ccRCC was considerably elevated. It appeared that ZFAS1 was consistently expressed at higher levels in ccRCC tumor cell lines than in the normal renal tubular epithelial cell line [16]. According to these findings, ZFAS1 acts as an oncogene in ccRCC. In particular, the interactions between ZFAS1 and miR-10a have been validated by luciferase reporter assay and RNA immunoprecipitation (RIP) assay [15]. MiR-10a silencing could attenuate the ability of ZFAS1 to promote ccRCC cell proliferation and metastasis. Subsequently, it was validated that SKA1, as a key downstream target protein for miR-10a, is responsible for the biological role of ZFAS1. ZFAS1 positively regulates SKA1 expression via miR-10a sponging. Through targeting the miR-10a/SKA1 pathway, ZFAS1 knockdown was shown to decrease the growth and metastasis of ccRCC in biological tests, suggesting that it may provide a new therapeutic target or biomarker for the disease [15]. Akin to MALAT1 above, lncRNA CYTOR was found to be upregulated while miR-136-5p was found to be downregulated in RCC cell lines and tissues [16]. CYTOR inhibition attenuated cell proliferation and invasion while promoting apoptosis. The lncRNA CYTOR sponged miR-136-5p, which negatively regulated RCC development. MiR-136-5p targets MAT2B. The corresponding MAT2B protein interacts directly with BAG3 protein to influence RCC cell proliferation, invasion, and apoptosis. In vivo experiments revealed that CYTOR knockdown increased miR-136-5p expression while decreasing MAT2B expression, thus preventing the development of RCC [16]. Several studies have concurred that CYTOR plays a crucial regulatory role in the initiation and growth of malignant tumors [17,18,19]. Compared to the control group, it was discovered that the expression of lncRNA CYTOR was raised in kidney tumor tissues and cells. Based on these findings, it has been suggested that lncRNA CYTOR may enhance RCC cell proliferation [16]. Previous studies have demonstrated that miR-136-5p participates in a number of physiological and pathological processes, including cell proliferation, differentiation, and apoptosis in a variety of malignancies, and it may help to inhibit cell growth [20,21,22]. These investigations demonstrated that RCC tissues and cells had lower miR-136-5p expression than the controls. Additionally, miR-136-5p binding to CYTOR was confirmed, and bioinformatics prediction software indicated target genes of lncRNA CYTOR. A functional experiment was carried out to learn more about the regulation of kidney cancer by the interaction of lncRNA CYTOR and miR-136-5p [16]. In particular, the results showed that lncRNA CYTOR knockdown inhibited renal cancer progression and could be reversed by miR-136-5p. These findings implied that CYTOR controlled miR-136-5p to affect the development of kidney carcinoma. Indeed, as mentioned earlier, by influencing the miR-136-5-p/MAT2B/BAG3 axis, CYTOR may contribute to kidney cancer [16]. According to one study, the CYTOR/miR-3679-5p/MACC1 axis may play a critical role in the development of CRC and carcinogenesis [23]. In addition, several studies have shown that lncRNAs ROR, UCA1, and MALAT1 may influence the growth of RCC cells differently. ROR promoted the progression of RCC cells through the miR-206/VEGF axis [24]; UCA1 influenced RCC cell growth through the miR-182-5p/DLL4 axis [25]; and MALAT1 sped up the development and progression of RCC cells by upregulating BIRC5 and decreasing miR-203 expression [26]. It has been shown that stimulation of the HIF1 pathway causes the production of H19, a lncRNA from a cluster of maternally imprinted genes on chromosome 11, in glioblastoma cell lines [27]. This discovery sparked a lot of curiosity to better understand how H19 functions in ccRCC and how the HIF pathway is upregulated. Compared to healthy kidney tissue, H19 was found to be more highly expressed in ccRCC [28]. H19 may also function in ccRCC cells as a ceRNA to prevent degradation of E2F1, a transcription factor that promotes cell proliferation [29]. Further research into several solid tumors revealed that H19 indeed interacted with a variety of transcriptional regulators and promoted the expression of genes related to EMT [30], thus highlighting H19 as a viable therapeutic target for cancer. In contrast to healthy kidney cells, ccRCC cells have notable overexpression of lncRNA HOTAIR, which is also linked to ccRCC progression [31]. As suggested by a recent study on the human HOX transcriptome [32], the carcinogenic potential of HOTAIR might depend on its interaction with PRC2 subunits, which mediates epigenetic reprogramming. 

**Figure 1:**
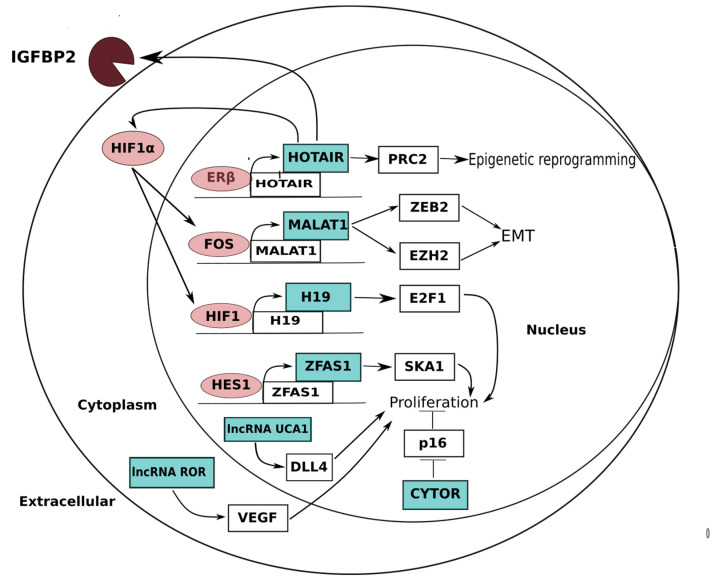
Summary of lncRNA oncogenic processes in renal cell carcinoma. In the hypoxia-inducible factor (HIF) pathway, which promotes angiogenesis, as well as other pathways that can increase tumor growth and therapeutic resistance in clear cell renal cell carcinoma (ccRCC), long non-coding RNAs (lncRNAs) play a significant role. The lncRNA HOX transcript antisense RNA (HOTAIR), the expression of which is positively regulated by estrogen receptor (ER), plays a crucial role in regulating ccRCC cell proliferation and angiogenesis by promoting expression of HIF1 and insulin-like growth factor-binding protein 2 (IGFBP2). HIF1 alone promotes the transcription of additional cancer-causing lncRNAs, including H1, which is an imprinted maternally expressed transcript that promotes tumor growth, and MALAT1, which promotes the activation of epithelial-mesenchymal transcription (EMT) programs. Other oncogenic lncRNAs include cytoskeleton regulator RNA (CYTOR), which suppresses P16 to encourage the growth of ccRCC, and ZFAS1, UCA1, and ROR, which induce tumor proliferation via miRNA sponging.

It has also been noted that HOTAIR may also affect other oncogenic pathways in ccRCC. In this context, it is intriguing that levels of insulin-like growth factor-binding protein 2 (IGFBP2), a protein that promotes proliferation, have been related to an increase in HOTAIR overexpression [33]. It is noteworthy that HOTAIR might function as a ceRNA to promote HIF1 expression in ccRCC cell lines [34]. Additionally, it has been shown that estrogen receptor (ER) is a prospective therapeutic target since it positively regulates HOTAIR expression in ccRCC [35]. The lncRNAs TCL6, NBAT-1, SPRY4-IT1, RCCRT1, GAS5, and CADM1-AS1 have all been associated with poor prognosis in patients with RCC [36,37,38,39,40] (Table 1, Figure 1). Approximately 15–20% of all renal malignancies are kidney renal papillary cell carcinomas (KIRP), and patients have a dismal prognosis. Treatment of KIRP is a significant clinical challenge because of its advanced stage at diagnosis and grim outcome [41]. Ferroptosis plays a role in pathological cell death in degenerative illnesses, in overcoming cancer cell resistance to chemotherapy, and in enhancing the clearance of faulty cells [42,43]. As an alternate treatment for cancer, ferroptosis provides a potential function as a tumor suppressor [44]. However, its impact on KIRP production through lncRNA regulation is unclear. As a result, co-expression analysis was used to investigate the connections between ferroptosis-related gene expression and lncRNAs.

Numerous lncRNAs have been linked to ferroptosis-related genes in KIRP [45]. Ferroptosis-related lncRNAs were split into high- and low-risk groups based on the risk score in order to examine their potential roles in KIRP. Prognosis-related lncRNA data were used to determine the confidence interval and hazard ratio [45]. Eight ferroptosis-related lncRNAs were discovered, which differed in expression between high- and low-risk patients and were associated with prognosis [45]. It was revealed that some lncRNAs were overexpressed in high-risk patients whereas others were overexpressed in low-risk cases (*p* < 0.05) [45]. The prognosis of patients with low-risk lncRNAs was better than that of patients with high-risk lncRNAs [45]. The lncRNAs CASC19, AC090197.1, AC099850.3, AL033397.2, LINC00462, and B3GALT1-AS1 were overexpressed in high-risk patients, suggesting that they can be considered cancer-promoting markers implicated in malignancy processes in KIRP patients. Recent years have seen the discovery of new regulators of ferroptosis, including the P53, ATF3/4, SLC7A11, ACSL4, and BECN1 pathways. LncRNAs are connected to controlling the expression of these factors [46].

By altering the P53 signaling pathway, ferroptosis-related lncRNAs have been found to affect the migration and proliferation of KIRP cells. A ferroptosis-related lncRNA prognostic model revealed that the low-risk subtype had a greater survival rate than the high-risk subtype. The idea has been used in a range of clinical contexts. These findings suggested that ferroptosis-related lncRNAs are vital biomarkers in predicting survival outcomes for KIRP patients [46]. As an anticancer therapy, a great opportunity is provided by the expansion of RNA-targeted treatments to modulate lncRNAs. Different lncRNA-targeting therapeutic methods are under investigation and many pharmaceutical companies are intensively working on developing lncRNA-targeted treatments [47,48]. Numerous studies have also demonstrated that the screening of suitable strategies for a notable modulation of lncRNAs depends critically on their cellular location [49]. While antisense oligonucleotides (ASOs) can deplete lncRNAs regardless of their location, small interfering RNAs (SiRNAs) can significantly decrease levels of cytoplasmic lncRNAs. LncRNA-targeting therapeutics can currently use ASOs [48].

## 3. Bladder Cancer

Up to 549,393 new cases of bladder cancer were reported worldwide in 2018 [50], making it a major cause of mortality globally. Both non-muscle invasive bladder cancer (NMIBC) and muscle-invasive bladder cancer (MIBC), which have different molecular patterns, are subtypes of bladder cancer. BlCa has a variable 5-year overall survival rate, ranging from 23% to 48% [51]. Currently, cystoscopy is the top diagnostic tool used for BlCa detection since carcinoma in situ is difficult to detect. In addition, the technique is invasive and occasional false negatives make matters worse [52]. Therefore, a number of screening tools, including computed tomography and radiographic imaging of the upper urinary tract, are necessary for the diagnosis of bladder cancer [53]. Patients with BlCa frequently have surgery, chemotherapy, radiation, and immunotherapy, but their prognosis remains poor and the 5-year overall survival rate is low [54,55]. Moreover, the combination of conventional chemotherapy and surgery is insufficient for the treatment of BlCa once the cancer has reached a locally advanced or metastatic stage. According to statistical data, the response rate to new immunotherapy with the use of immune checkpoint inhibitors (ICI) is 30% or less [56]. Due to the limitations in BlCa treatments, new therapeutic targets are needed to improve the patient survival time. Due to a combination of the above mentioned factors, classifying the risk of BlCa patients and making accurate treatment decisions has been challenging for urologists [57]. Therefore, there is an urgent clinical need to find molecular biomarkers for BlCa via molecular profiling. The high frequency of genetic alterations is one of the crucial factors in the context of BlCa. One of the most frequent mutations observed in over 70% BICa patients is in the gene encoding telomerase reverse transcriptase (TERT) [58,59,60,61]. It is thus imperative to pinpoint the chemical elements contributing to the development of BlCa. Several studies have focused on identifying the causes of BlCa progression and therapeutically addressing them. Many molecular pathways are affected in BICs and their functional restoration could pave the way for an effective eradication of this tumor [62,63,64,65]. Indeed, several countries have initiated BICa genetic profiling. 

For instance, P53, Bcl-2, Bax, BMI1, and CD44 are among the variables that exhibited aberrant expression in Iranian patients with BlCa [66]. Such investigations are crucial for developing precision medicine-based therapies. Genetic and epigenetic mechanisms also control BlCa cell migration, proliferation, and treatment response. Non-coding RNAs (ncRNAs) have attracted considerable attention as epigenetic regulators of BlCa development [67]. Since it is known that ncRNAs can control a variety of biological processes [68,69], it follows that they have a significant impact on the growth, migration, and therapeutic response of BlCa [70,71]. The first consideration of lncRNA functions in BlCa may be connected to growth and viability. It has been shown that colon cancer-associated transcript 1 lncRNA (CCAT1) is overexpressed in BlCa and has favorable correlations with cancer stage, size, and grade.

Targeting the lncRNA CCAT1 may offer a new tool for BlCa therapy [72]. The conversion of BlCa cell metabolism to aerobic glycolysis is a well-known method by which cells multiply more quickly [73]. Thus, the discovery of the mechanisms underlying the metabolic reprogramming of BlCa cells may open the door to therapeutic intervention. According to many reports, the development of BlCa correlated with the induction of aerobic glycolysis and upregulation of hypoxia-inducible factor-1α (HIF-1α) and β-arrestin 1 proteins [74,75]. Clinical investigation also demonstrated a link between enhanced glycolysis and worse outcomes for BlCa patients. LncRNAs are thought to control glycolysis in BlCa. As BlCa progresses, lncRNA SLC16A1-AS1 can promote glycolysis and mitochondrial respiration by increasing ATP generation. Therefore, lncRNA SLC16A1-AS1 promotes BlCa proliferation by increasing β-oxidation of fatty acids [76]. Targeting these lncRNAs could inhibit aerobic glycolysis in BlCa cells and thus impair their proliferation.

Apoptosis is another feature of BlCa cells that lncRNAs can control. An example of how lncRNAs negatively regulate apoptosis in BICa is provided by SNHG7. The latter is highly expressed in BICa. Once its expression is suppressed by siRNAs, apoptosis ensues [77]. Another lncRNA, ZEB2-AS1, promotes BlCa cell proliferation and inhibits apoptosis [78]. LncRNAs are also known to control the cell cycle progression of cancer cells through cyclin-dependent kinase 9 (CDK9). Indeed, reduced expression of CDK9 leads to cell cycle arrest and apoptosis [79]. Through the overexpression of CDK9, lncRNA GAS6-AS2 encourages the proliferation and viability of BlCa cells. In contrast, cell cycle arrest at the G1 phase has been linked to the silencing of GAS6-AS2, which downregulates CDK9 expression [80]. CDK1 is another modulator of the cell cycle. The progression of BlCa cells is hampered when CDK1 is downregulated by tristetraprolin [81].

In addition, licochalcone B induces cell cycle arrest in BlCa cells by inhibiting CDK1 [82]. It was discovered that lncRNA PVT1 increased the viability and proliferation of BlCa cells by upregulating CDK1 [83]. Moreover, lncRNAs are associated with the stemness of BlCa cells. In the initial stage, NCK1-AS1 promoted BlCa development and was linked to an increase in the abundance of CD133+ stem cells [84]. Since lncRNAs regulate cell cycle progression, apoptosis, and stemness, targeting them may result in decreased viability and survival rates for BlCa cells.

Studies on extracellular vesicles (EVs) [85,86] have shown that these tiny structures, which are enclosed by a lipid bilayer membrane, play crucial roles in biological processes depending on the load they carry, which can include lipids, proteins, or nucleic acids [87,88,89,90,91]. Through exocytosis, exosomes are secreted into the extracellular milieu and are thought to play a role in cell-cell communication [92]. Exosomes have been shown to transport lncRNAs between cells, which can affect tumor cell growth, invasion, and treatment resistance [93]. Studies have concentrated on the function of exosomes in the transport of lncRNAs and the connection to BlCa. It has been demonstrated that exosomal release of the lncRNA PTENP1 causes apoptosis in BlCa cells and inhibits its development. Moreover, PTENP1-containing exosomes are released from normal cells to deliver this antitumor factor to BlCa cells [93].

Numerous studies have demonstrated the important role played by immune-related long noncoding RNAs (IRLs) in the tumor microenvironment (TME) [94]. Many cancers have a higher risk associated with gene polymorphisms in lncRNAs [95,96]. A signature consisting of four lncRNAs (HCP5, IPO5P1, LINC00942, and LINC01356), all of which were discovered for the first time in bladder cancer, was established. Compared to the typical clinical features of BlCa patients, the IRL signature had a strong predictive ability for overall survival (OS) [97]. Surprisingly, the TME immune cell infiltration and ICI therapy response had a strong association with the IRL signature. Therefore, in comparison with earlier data, this novel IRL signature seems to have a superior role in predicting patient prognosis and the effectiveness of ICI immunotherapy [97]. 

Reducing cell proliferation and invasion may not be sufficient for a recovery from BlCa. It has recently been demonstrated that BlCa cells can become resistant to therapy by activating tumor-promoting factors such Akt, autophagy, and integrin 8 [98,99,100]. This calls for new therapeutic strategies designed to minimize drug resistance. Indeed, the evolution of resistance makes cisplatin (CP) treatment ineffective for treating BlCa patients. It is hypothesized that the lncRNA MEG3 enhances CP cytotoxicity against BlCa cells. MEG3 reduces the expression of matrix metalloproteinase-2 (MMP2) and MMP9 in BlCa cells, which lowers their ability to invade. It appears that upregulating P53 and boosting the production of pro-apoptotic proteins such as Bax and caspase-3 are two ways through which MEG3 could make BlCa cells more susceptible to CP-mediated apoptosis. Additionally, MEG3 inhibits Bcl-2 expression to cause cell death [101]. The lncRNA DLEU1, on the other hand, acts in a reverse manner and promotes BlCa cell migration and proliferation. DLEU1 is highly expressed in BlCa and associated with poor prognosis. By blocking miRNA-99b, DLEU1 mechanically increases HS3ST3B1 expression and causes CP resistance [102]. LncRNAs can control P53, which in turn affects how CP affects BlCa cells. By inhibiting acetylation, sirtuin 3 (SIRT3) can decrease P53 expression [103]. An increase in the expression of lncRNA MST1P2 leads to CP resistance. MST1P2 inhibits miRNA-133b to increase SIRT3 expression, which in turn downregulates P53 and contributes to drug resistance [104]. The lncRNA UCA1 stimulates the production of Wnt6, which is also closely related to CP resistance. UCA1 silencing leads to inactivation of Wnt signaling and consequently to suppression of CP drug resistance [105]. Interestingly, UCA1 can make BlCa cells resistant to both CP and gemcitabine [106]. H19 is another key lncRNA that is overexpressed in BlCa [107,108,109] through the induction of EMT by multiple mechanisms. Interactions with transcriptional regulatory complexes are necessary for one of these mechanisms. For instance, H19 binds to the EZH2 subunit of PRC2, which promotes WNT-β-catenin pathway activation and consequently the EMT [30,110]. Additionally, it has been shown that H19 functions as a ceRNA to prevent miRNAs from degrading the essential transcriptional regulator DNA (cytosine-5)-methyltransferase 3B (DNMT3B), which is connected to the development of EMT in cancer [111]. It is interesting to note that the expression of H19 in BlCa is increased by the EMT protein YAP1 [112]. As mentioned earlier, the expression of MALAT1, an important epigenetic regulator implicated in oncogenic pathways in a range of tumor types, is dysregulated in bladder cancer. The TGF pathway [113], which promotes the activation of transcriptional programs that support EMT, also seems to include MALAT1 as a downstream target. The EZH2-mediated transcriptional regulation found in prostate cancer [114] and ccRCC [11,14] appears to be different from this mechanism. By directly interacting with the PRC2 protein SUZ12, MALAT1 functions as a transcriptional regulator in the context of bladder cancer [115]. By boosting the expression of transcription factors ZEB1, ZEB2, and zinc finger protein SNAI2, which promote EMT, MALAT1 is specifically conducive to EMT [116]. 

Overall, lncRNAs can influence miRNA expression directly or indirectly. LncRNAs directly lower miRNA expression through sponging. Indirectly, they alter the level of expression of miRNAs by attracting other factors, such as EZH2, to act as their promoters [68,117,118]. To increase the expression of miRNA-196a-5p in BlCa cells, lncRNA UCA1 employs CREB as a transcription factor for the miRNA-196a-5p promoter. Consequently, UCA1 confers resistance to cisplatin/gemcitabine in bladder cancer cells by activating miR-196a-5p via CREB [106]. 

**Figure 2:**
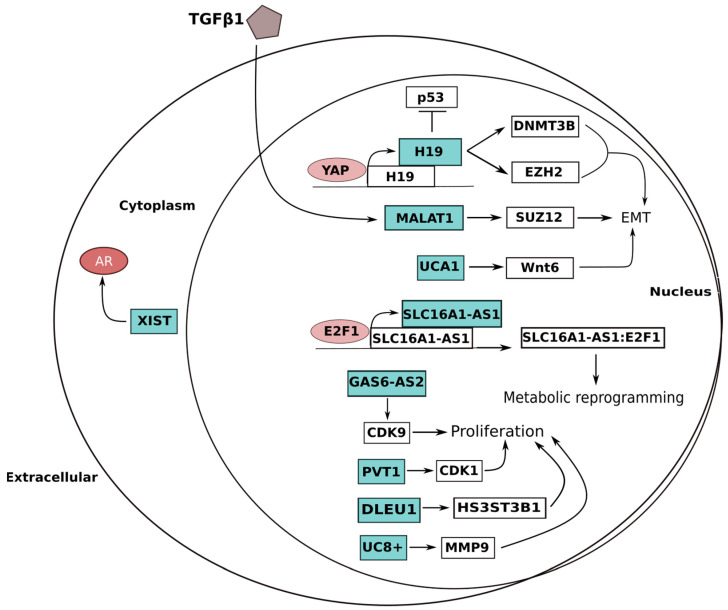
Summary of lncRNA involvement in bladder cancer (BlCa). LncRNAs are essential for the invasive features of BlCa in order to promote metastasis and dissemination. They also take part in numerous other oncogenic pathways that might advance its progression. Many of these processes are influenced by urothelial cancer-associated 1 (UCA1), which specifically recruits CREB to the miRNA-196a-5p promoter as a transcription factor. By increasing several EMT-activating transcription factors, such as zinc-finger E-box-binding homeobox 1 (ZEB1) and 2 (ZEB2), twist-related protein 1 (TWIST1), and zinc-finger proteins SNAI1 and SNAI2, the lncRNA MALAT1 encourages EMT. The transforming growth factor-β1 (TGFβ1) pathway positively controls MALAT1. By upregulating DNA (cytosine-5)-methyltransferase 3B (DNMT3B) and histone-lysine N-methyltransferase EZH2 and inhibiting TP53, the H19 imprinted maternally expressed transcript also promotes EMT. In order to affect cancer metabolic reprogramming toward the formation of a hybrid oxidative phosphorylation/glycolysis cell phenotype favoring BlCa invasiveness, the lncRNA-SLC16A1-AS1 complex with its transcription factor is required. The lncRNAs GAS6-AS2 and PVT1 promote tumor growth by upregulating cyclin-dependent kinases. Via miRNA sponging, the lncRNAs DLEU1 and UC8+ promote cell proliferation. By increasing androgen receptor (AR) signaling, X inactive specific transcript (XIST), in contrast to its role in prostate cancer, may have an oncogenic role in BlCa. The figure highlights simplified interactions between lncRNAs and carcinogenic pathways and does not adequately depict the subcellular location of lncRNAs.

Additionally, the lncRNA containing uc.8+, which is a member of the class of lncRNAs known as transcribed ultraconserved regions (T-UCRs), is the most upregulated T-UCR in BlCa [119]. Specifically, a previous qPCR-based expression analysis [120] conducted on RNA isolated from BlCa biopsies (*n* = 40) [121] revealed that uc.8+ levels correlated with both grading (i.e., cell differentiation) and staging (i.e., tumor invasiveness), although its expression was lower than in precancerous bladder tissues [122]. It is intriguing to note that in contrast to other malignancies, a number of lncRNAs may potentially have special functions in bladder cancer. For example, XIST has been characterized as a tumor suppressor in prostate cancer, but it functions as a ceRNA in bladder cancer and increases AR signaling, an important pathway in tumor development and drug resistance [123]. Fluorescence microscopy data for BlCa cell line J82 showed that uc.8+ was a natural decoy for miR-596, thus the upregulation of uc.8+ caused MMP9 to be expressed more frequently, which increased the ability of bladder cancer cells to invade other tissues (Table 2, Figure 2). Since BlCa patients have a poor prognosis, lncRNA targeting can be viewed as a potentially successful treatment option [124]. Furthermore, these patients frequently experience chemotherapeutic failure. Synergistic therapy can be achieved by combining genetic tools and anticancer agents. Notably, erdafitinib was the first targeted treatment for metastatic bladder cancer to receive approval [125].

The FDA recently approved the use of combination therapies using ICI and targeted drugs, such as pembrolizumab or avelumab with axitinib, because they have been proven to be efficient and safe [126].

Interestingly, there is no evidence linking lncRNAs to the response of BlCa cells to radiotherapy. Studies are needed to identify the lncRNAs involved in radiotherapy resistance or sensitivity because it is a key component of BlCa therapy. As previously mentioned, exosomes can contain lncRNAs in BlCa and may affect the development of BlCa by transferring ncRNAs. The role of exosomal lncRNAs in BlCa development needs more research as well. Finally, a better understanding of the interaction between lncRNAs and MMP and the effects on BlCa cell migration and invasion would be beneficial to design novel therapies.

## 4. Prostate Cancer

Pca is the second most frequent cancer among older men, which also rates as the second leading cause of cancer-related mortality [5]. The incidence and death due to this tumor have significantly increased over the past ten years due to changes in aging, lifestyle, and the environment [127]. Patients who undergo close monitoring and endocrine therapy at a very early stage might retain a generally favorable prognosis within 5 years; however, in many patients, it is detected at middle or late stages due to hidden symptoms [128,129,130]. Currently, in accordance with the conventional mechanism of the androgen receptor (AR) signaling pathway, androgen deprivation therapy (ADT) is the first-line systemic treatment for advanced PCa [131]. However, it has been found that the innate cellular diversity in PCa can eventually adapt to ADT. Castration-resistant prostate cancer (CRPC) is a severe condition that can develop as a result of this activation of AR signals, even at low blood androgen levels [132,133]. Serum testosterone values less than 50 ng/mL or 1.7 nmol/L, along with biochemical or radiographic progression, make up the diagnostic criteria used to prognosticate CRPC [134]. According to the guidelines of the Response Evaluation Criteria in Solid Tumors, the presence of more than two new lesions found during an imaging survey is specifically referred to as radiological progression (RECIST) [135]. After initial PCa treatment, the prevalence of CRPC is estimated to be 10–20% within 5 years of follow-up, with metastatic CRPC (mCRPC) representing 84% of all cases. Furthermore, 33% of non-metastatic CRPC cases will develop metastases within 2 years of follow-up [136]. The median PCa-specific survival in mCRPC only increases by 2.8 to 4.8 months since the mechanism of CRPC is highly complex and changeable [137,138,139,140,141,142,143]. This is true even with novel medications continually being proposed and employed. The main mechanism of CRPC is believed to be the persistent activation of AR signaling in cancer cells, even in the presence of ADT [144]. Nevertheless, the majority of CRPC cases are dependent on AR signaling because it occurs via a number of additional routes. These mechanisms can include AR point mutations, increased AR expression, altered intratumoral androgen biosynthesis, emergence of AR splice variants, as well as cofactor activity modulations [145]. Abiraterone, apalutamide, and enzalutamide are examples of more potent AR signaling inhibitors (ARSI) that are acknowledged as first-line therapeutic alternatives in CRPC [146]. However, not all castration resistance is dependent on AR signaling because of the tumor’s variety, heterogeneity, and flexibility. Further resistance to ARSI may result from tumor cells switching from AR-dependent to AR-independent signaling pathways [147,148]. It has been established that ncRNAs, which participate in a variety of molecular regulatory activities, including signal transduction, post-transcriptional regulation, post-translational regulation, and epigenetic regulation, are an essential molecular component in many of the pathologic mechanisms underlying CRPC [149,150,151]. The two ncRNA categories that are most frequently studied in CRPC research are miRNAs and lncRNAs, both of which have been shown to be involved in a variety of patho-physiologic pathways [150,151]. The first function of miRNAs in CPRC was shown to be regulatory. CPRC has been linked to the formation of at least 20 different types of miRNAs, which take part in a variety of pathogenic pathways, such as AR-related cell proliferation, cancer cell survival, apoptosis, or EMT [149]. On the other hand, because of advancements in detection technology over the past ten years, the connection between CPRC and lncRNAs, as larger and more complex ncRNAs, has been steadily investigated [150].

A single type of lncRNA can also be regulated by many pathways due to the fact that lncRNAs contain more information and have a stronger affinity for biomolecules in the human body. As a result, lncRNAs play important regulatory roles in the development of PCa from an androgen-dependent state to CRPC [152]. For instance, the AR protein may upregulate AR-regulated long noncoding RNA 1 (ARLNC1), which then stabilizes the AR transcript by RNA-RNA interaction [153]. In vitro and in vivo AR-dependent PCa cell growth was inhibited by ARLNC1 knockdown, which also resulted in decreased AR expression [152]. Prostate cancer-associated long-noncoding RNA 1 (PRNCR1) and PCa gene expression marker 1 (PCGEM1) are highly expressed in CRPC and known to be involved in the AR signaling pathway; however, knockdown of either PRNCR1 or PCGEM1 inhibited the growth of CRPC cells in vivo [154,155,156]. Interactions with miRNAs provide another method via which lncRNAs can affect AR.

Growing evidence suggests that the interaction patterns between lncRNAs and miRNAs are strongly linked with the development of cancers [157,158]. It has been demonstrated that they interact in a variety of ways, including the most popular “sponge” effect [159,160]. Over the past five years, several studies have outlined regulation mechanisms and interactions between lncRNAs and miRNAs in several CRPC pathogenic pathways [161]. The critical role of tyrosine kinase receptors (RTKs) has also been defined, as well as how activation of the downstream signaling cascade can give rise to the changing phenotypic and molecular landscape of metastatic CRPC [161]. 

Liu et al. assessed the expression of lncRNA AFAP1-AS1 in castration-resistant C4-2, PC3, and DU145 cell lines, discovering that their expression was considerably higher than in androgen-sensitive LNCaP cells. To suppress IGF1R, AFAP1-AS1 binds to miR-15b and interferes with its tumor suppressor function [162]. According to Huang et al., lncRNA PTTG3P levels were noticeably higher in CRPC tissues and castration-resistant cell lines. By competing for miR-146a-3p, PTTG3P may enhance PTTG1 expression [163]. SChLAP1, another lncRNA, is significantly highly expressed in PCa and is intimately associated with tumor development. SChLAP1 has the potential to suppress the expression of miR-198, which is specifically correlated with the 3′ UTR of MAPK1.

The principal target genes of miR-198, including ELK-1, F-actin, and PAK1, are associated with the advancement of cancer [164] and miR-198 suppression greatly enhanced the expression of MAPK1.

The lncRNA Linc00963 was shown to be upregulated in C4-2 cells, as opposed to LNCaP cells, highlighting its function in the change from androgen-dependent PCa to androgen-independent CRPC [165]. Linc00963 is directly attached to miR-655 in CRPC cells, and prevents it from interacting with TRIM24 mRNA. This promotes cell proliferation by increasing TRIM24 [166]. In advanced CRPC, TRIM24 is a well-known oncogene [167]. The activation of AR by TRIM24 expression via the PI3K/AKT pathway may aid in the prevention of CRPC. Additionally, it was shown that TRIM24 controls the transcription of the EGFR and PIK3CA genes, and that PIK3CA and EGFR work in synergy to activate the PI3K/AKT pathway in PCa [167]. The lncRNA SNHG7 is overexpressed in PCa tissue and cell lines and strongly associated with poor prognosis. Its knockdown reduced critical cell cycle regulators, such as cyclin D, CDK4, and CDK6. Both in vivo and in vitro studies have shown cell cycle arrest in the G0/G1 phase following SNHG7 silencing [168]. Furthermore, it was discovered that miR-503 had complementary binding sites within the 3′UTRs of both SNHG7 and cyclin D. Thus, miR-503 mimic transfection has been shown to reduce cyclin D and SNHG7 miRNA expression and inhibit tumor growth [168]. The PCa cell’s cytoplasm is home to lncRNA LOXL1-AS1, which acts as a miRNA sponge to interact with miR-541-3p [168]. By interacting with the 3′UTR of cyclin D, this miRNA suppresses its expression and causes G0/G1 phase cell cycle arrest. Genes highly enriched in nuclear division and cell cycle checkpoints were upregulated by LOXL1-AS1 induction. Through modifying the expression of miR-541-3p and subsequently cyclin D, LOXL1-AS1 controlled cell cycle progression [169]. 

In PCa, lncRNAs can also control apoptosis. An apoptosis-related mechanism involving toll-like receptor (TLR) is induced in the TME [170]. The activation of TLR signaling pathway accelerates PCa progression [171]. The lncRNA PART1 can activate TLR signaling and its downstream targets, such as TLR3, TNFSF10, and CXCL13, to suppress PCa cell death. PCa growth and apoptotic induction are both reduced when PART1 is silenced [172]. Similarly, by decreasing the expression of miRNA-15a-5p, the lncRNA PVT1 encourages KIF23 expression to inhibit apoptosis in PCa cells. Indeed, apoptosis induction and PVT1 knockdown have been shown to be linked [173]. 

Metastasis, which occurs when cancer cells migrate to distant organs, such the lung, liver, bone, and lymph nodes, is a major cause of PCa-related mortality [174]. Bone metastasis, the most frequent side effect of PCa, is consequently linked to osteoblastic and osteolytic diseases [175]. Therefore, it is essential to pinpoint the causes of PCa metastases in order to effectively treat this dangerous condition. Molecular pathways linked to metastasis can also be thought of as prognostic biomarkers [176,177]. The NDRG1 gene, the downregulation of which increases migration, is one of the molecular processes involved in controlling PCa metastases [178]. As a tumor suppressor factor, the lncRNA LINC00844 is downregulated in metastatic PCa and is linked to poor prognosis. In order to promote NDRG1 expression and prevent PCa cell migration and invasion, LINC00844 mechanically mediates the binding of AR to chromatin [179]. C-X-C chemokine receptor type 4 (CXCR-4) is another component that contributes to PCa bone metastases [180,181]. CXCR4 is overexpressed in many cancers and hence responsible for their aggressive behavior [182,183,184,185]. Its overexpression in PCa causes lymph node and bone metastases and is linked to poor prognosis [186]. The lncRNA UCA1 can control CXCR4 expression in PCa to influence tumor development. The former stimulates the production of the latter to increase metastasis of PCa by sponging miRNA-204 [187]. Overall, lncRNAs have been shown to be important regulators of PCa metastasis, and more work is ongoing to identify other lncRNAs involved in facilitating PCa cell migration and invasion [188,189]. Indeed, the lncRNA ATB is a tumor-promoting factor that promotes PCa cell proliferation and invasion, and its overexpression is associated with poor prognosis [190]. Because lncRNAs can help PCa evade the immune system, their expression levels affect how well PCa responds to immunotherapy [191]. As expected, tumor-suppressor lncRNAs experience considerable downregulation, in contrast to tumor-promoting lncRNAs, which are highly expressed in PCa. The lncRNA TINCR has been linked to clinical T staging, lymph node change, and distant metastases in PCa. Because low TINCR expression indicates poor prognosis, it is crucial to include evaluation of its expression levels in clinical courses [192]. PCa patients with low expression of tumor suppressor lncRNA DGCR5 have a worse chance of survival [193]. As a result, identifying these lncRNAs and determining their degree of expression can be used as an effective and trustworthy predictive tool [194]. Additionally, the serum of PCa patients can be tested for expression levels of exosomal lncRNAs as a diagnostic and predictive tool [195]. However, lncRNAs in PCa can also be targeted with anticancer drugs.

Since most antitumor agents in nature that target lncRNAs are phytochemicals and have poor bioavailability, approaches such as using drug delivery vehicles to increase potency should be taken into consideration [196]. A natural substance of plant origin called quercetin is frequently used in PCa therapy. The response of PCa cells to chemotherapy can be greatly enhanced by the ability of quercetin to inhibit cell migration and proliferation. Additionally, nanoparticles have been created for the release of quercetin to enhance its anticancer action against PCa [197]. It targets a number of lncRNAs. In a concentration- and time-dependent manner, quercetin lowers the expression level of MALAT1. In addition to the in vitro study, in vivo experiments with xenograft tumors demonstrated the effect of quercetin in preventing the spread of PCa. Through the regulation of EMT, quercetin prevented metastases by downregulating MALAT1. Quercetin negatively affects cell proliferation by inhibiting the PI3K/Akt pathway [198]. 

**Figure 3:**
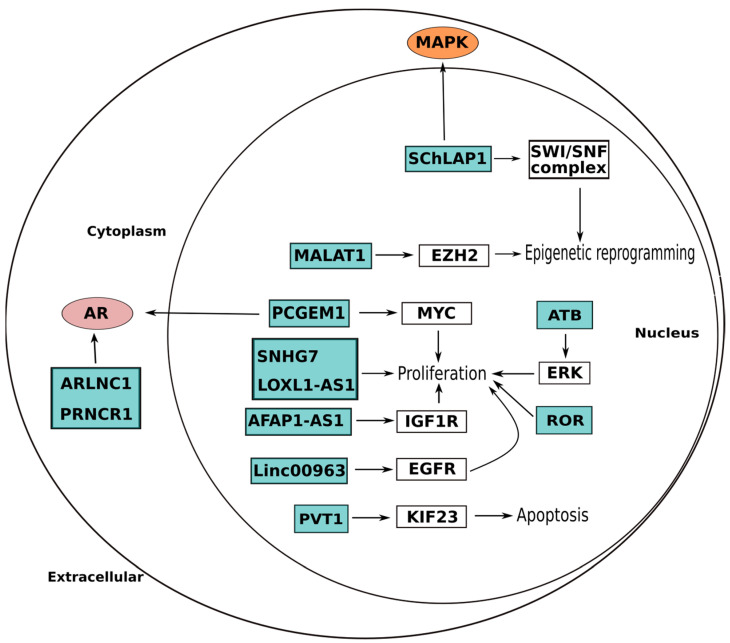
LncRNAs and their role in prostate cancer (PCa). Numerous lncRNAs promote the advancement of androgen receptor (AR)-dependent PCa by upregulating AR, stabilizing the transcript, and stabilizing the protein. Prostate-specific transcript (PCGEM1), prostate cancer-associated non-coding RNA 1 (PRNCR1), and androgen receptor-regulated long non-coding RNA 1 (ARLNC1) are notable examples. In particular, mitogen-activated protein kinase (MAPK) signaling is one of the numerous AR-independent pathways that is supported by lncRNAs, such as the SWI/SNF complex antagonist linked with prostate cancer 1 (SChLAP1). The promotion of survival and proliferation through MYC activation (PCGEM) and epigenetic reprogramming via chromatin remodeling complexes are two important cellular processes that can be altered by SChLAP1 and MALAT1. The lncRNAs SNHG7 and LOXL1-AS1 induce cancer proliferation by miRNA and cyclin regulation. The lncRNAs ATB and ROR induce cancer proliferation and invasion by regulating the PI3K\Akt pathway. LncRNAs AFAP1-AS1 and Linc00963 accelerate cancer proliferation. The figure has been simplified to improve readability and hence the representation of lncRNAs and oncogenic pathway connections may not accurately reflect their subcellular localization.

Another well-known naturally occuring anticancer substance is curcumin. It is derived from the rhizome and root of the *Curcuma longa* plant. Curcumin slows the growth of PCa by triggering apoptosis and cell cycle arrest by controlling NF-B signaling and preventing angiogenesis [199]. PCa stem cells are adversely affected by curcumin administration, which inhibits their migration and proliferation. The lncRNA ROR functions as a ceRNA to decrease miRNA-145, thus advancing PCa [200]. Interestingly, curcumin administration decreases ROR expression while increasing miRNA-145 expression and limits PCa cell proliferation [200] (Table 3, Figure 3). 

In conclusion, lncRNAs can control the growth and metastasis of PCa cells. Additionally, they can program cell death by modulating autophagy and apoptosis in PCa. LncRNAs have several downstream targets; STAT3, NF-B, PTEN, PI3K/Akt, and miRNAs being some of the most significant ones [201]. Generally, expression of tumor-promoting lncRNAs is increased in PCa and that of tumor-suppressor lncRNAs is decreased. In addition, lncRNAs can control how PCa cells react to chemotherapy and radiotherapy. Some lncRNAs are also responsible for chemoresistance to PTX and DOX. A reduction of such lncRNA may improve the effectiveness of anticancer chemotherapy [201]. Additionally, via modulating radio resistance, lncRNAs can encourage the suppression of autophagy. To assess their pro-survival and pro-death roles in PCa, the link between the two needs to be further studied. Cytotoxic T cells are essential for stopping the spread of PCa by activating antitumor immunity. Therefore, in order to increase the potential of immunotherapy lncRNAs with a positive influence on this T cell subset are urgently needed to be identified. Thus, lncRNAs seem to play a crucial role in PCa suppression, and pharmacological and genetic therapies are in the offing to target them. Furthermore, lncRNAs can also be employed as diagnostic and prognostic tools for PCa patients in clinical settings [201]. In order to pave the road for PCa treatment, future research will need to concentrate on identifying more lncRNAs implicated in the disease’s progression or suppression.

## 5. Other Genitourinary Cancers

Other genitourinary cancers include ovarian cancer (OC), one of the main common gynecological cancers. Globally, there were over 295,000 new OC cases in 2018, among which 184,000 were fatal [51]. Epithelial OC (EOC), which accounts for over 90% of cases, has four main histological subtypes: serous, endometrioid, clear cell, and mucinous. Their primary sites of origin are the fallopian tube and endometrium, with ovary involvement occurring secondarily [202]. However, the asymptomatic character of early-stage disease, different histological subtypes, and resistance to therapy continue to be significant contributors to poor prognosis and low 5-year overall survival rates (40%) [203,204]. Advanced OC (AOC) is defined as stage FIGO IIB to IV, where cancer cells have metastasized to the peritoneal cavity and exhibit resistance to first-line therapy. Stage I-IIA is restricted to the ovaries [205]. Cytoreductive surgery is the first step in treating OC, followed by platinum- and taxane-based chemotherapy. First-line treatments often work well for patients with e I–IIA stage OC. However, unfortunately, the disease returns in more than 85% of AOC patients who receive the standard treatment and achieve complete remission during the first two years, with a median survival of less than 24 months [203,206]. Recurrent OC (ROC) is characterized by a small number of OC cells that have undergone therapeutic stress and repopulate the site of origin or secondary metastatic locations. For OC patients, the poor prognosis of ROC remains a substantial management problem. Cancer stem cell (CSC) activity, invasion, and metastatic features, and treatment resistance are all linked to ROC progression [207]. Therefore, it is crucial to find novel therapeutic approaches and diagnostic/prognostic markers. 

Dysregulation of lncRNA is a significant factor in the early onset, development, spread, and chemoresistance of ROC [208,209,210]. The differential expression of H19, LSINCT5, XIST, CCAT2, HOTAIR, AB073614, and ANRIL is linked to oncogenic and invasive characteristics of OC cells [211,212,213]. More than 500 differentially expressed lncRNAs between original tumor cells and malignant OC cells from ascites have been reported [214]. Additionally, their variable levels after treating OC with carboplatin-docetaxel suggest a role in predicting medication response [215]. Targeting particular processes in OC progression has been made possible by the functional involvement of lncRNAs with various mechanisms of action, in particular with OC stages [216]. Cancer metastases and the stem-like characteristics of cancer cells are both directly related to EMT [217]. ROR, HOTAIR, H19, and UCA1 encourage EMT and control the proliferation of CSCs [218,219]. Having stem cell markers, including CD24, CD44, CD133, CD44v6, NESTIN, NANOG, and OCT3/4, that reflect self-renewal capabilities and propensity to spread, CSCs are crucial in predicting metastasis, chemoresistance, and relapse [220,221,222]. The biomarker Ca-125, also known as MUC16, is frequently evaluated in serum to assess the progression and response of OC to treatment [223]. However, its applicability in predicting the prognosis of OC patients is constrained by variability in Ca-125 expression levels in later stages of the disease and correlation with other clinical conditions. In precision medicine, liquid biopsy-based disease screening and detection methods have become more common [224]. Several techniques, including quantitative reverse transcription-polymerase chain reaction (qRT-PCR), fluorescent in situ hybridization (FISH), microarray hybridization, and gene profiling, have been used to evaluate the expression of lncRNAs involved in the proliferation, apoptosis, invasion, and migration of tumor cells [225]. The expression of ANRIL, HOTAIR, MALAT1, and UCA1 in OC cells was found to be histotype-specific and thus identifying them as potential detection/diagnostic biomarkers. Due to its higher expression in ROC relative to normal tissue, ANRIL has the potential to be employed as a biomarker to predict poor survival in patients with ROC [226]. The differential expression levels of circulating extracellular lncRNAs connected to bodily fluids, such as plasma/serum, may serve as biomarkers for routine clinical use to predict the prognosis, risk of tumor spread, and recurrence after surgery [227]. The capacity of circulating lncRNAs to withstand RNase makes them excellent biomarkers [227]. Therefore, the use of circulating lncRNAs as tumor biomarkers may be clinically beneficial because of their high sensitivity, specificity, and due to non-invasive mode of collection. It has been suggested that the circulating lncRNAs H19, HOTAIR, and MALAT1 in bodily fluids, such as plasma, may function as indicators of drug response, risk of tumor metastasis, and recurrence in OC patients after first-line therapy [227].

The upregulation of ANRIL, AB073614, CCAT2, HOTAIR, NEAT1, TC0101441, and UCA1 in primary samples was also positively correlated with poor prognosis for OC patients [228]. It has been proposed that C17orf91, also known as MIR22HG, can be employed as a predictive biomarker for OC patients due to its increased expression between primary and metastatic tumors [229]. Furthermore, since lower GAS5 expression indicates poor prognosis, growth-stop-specific 5 (GAS5), a tumor suppressor lncRNA in OC, may be useful for assessing the patient’s response to first-line therapy [230]. Increased lncRNA MEG3 expression inhibits proliferation and induces apoptosis by upregulating miRNA and expression of TP53, GDF15, and RB1. MEG3 has been reported to be suppressed by promoter hypermethylation in the majority of EOC tissues [231]. Increased SNHG15 expression has been shown to play a critical role in EOC migration, invasion, proliferation, and chemoresistance and has been linked to pathophysiology, ascites, and higher FIGO stage [232].

In EOC, a prognostic indicator and potential target are both possible due to the association between the high expression of SNHG15 and resistance to cisplatin [232]. These findings imply the potential utility of lncRNA expression profiles in screening, illness monitoring, and as prognostic indicators in OC [233]. Targeting lncRNAs for therapy may be a promising strategy because of their highly potent and particular functional importance in OC development. The following methods can be used to target them: (1) using siRNA or antisense oligonucleotides (ASO) with chemical modifications to degrade post-transcriptional RNA; (2) inducing loss of function by producing steric inhibition of RNA-protein interactions by ASO; (3) preventing interactions between lncRNAs and miRNAs using oligonucleotides; and (4) modifying lncRNA expression by promoting steric hindrance [233]. Great strides have been made in understanding the roles of lncRNAs in OC, but further studies are needed for their therapeutic applications. It is crucial to find the most therapeutically relevant candidates in ROC that have cancer-enriched or cancer-specific signatures and can predict or identify the early stages of disease. The development of lncRNA-based techniques can be greatly sped up by identifying the expression signatures and predicting target sites in silico utilizing reliable algorithms or bioinformatics tools [233].

The development of nanotechnology-based sensors and nanoscale delivery vehicles can improve the success of clinical translations of lncRNA-based techniques. It is suggested that future research should focus on developing cutting-edge nanoparticle-based methods to block their functions associated with drug resistance and spread in ROC. Although promising, the use of ASOs is currently limited by poor membrane permeability, which prevents nuclear lncRNA targeting [233]. Additionally, the stability and permeability of ASOs or other lncRNA targeting agents can be improved with nanoparticles, allowing them to reach the desired regions. In summary, more efforts are urgently needed to determine whether and how lncRNA-based technologies can be clinically useful in ROC [233].

Another genitourinary cancer is testicular germ cell tumor (TGCT), a common solid tumor in young men aged 20–40 and is the leading cause of death from solid tumors in men of this age. TGCT is classified into two types: seminoma and non-seminoma. The global incidence of TGCT is increasing [234,235], with 15–30% of patients experiencing recurrence and metastases, frequently associated with poor prognosis [236]. Researchers have discovered a range of increased gene expression in TGCT in recent years. BOB1 and prominin 1, two new germ cell markers, are upregulated in seminoma [237]. Cyclin D2 and N-Myc were overexpressed in rat spermatogonial cells by Houldworth et al. [238]. Cyclin D2 is a precursor to carcinoma and is crucial for the development of germ cell malignancies [239]. These findings show that TGCTs are associated with aberrant gene expression patterns and that different testicular tumor subtypes have distinct regulatory mechanisms for genes involved in processes such as proliferation, pluripotency, and epigenetics. More research is required to determine how these gene targets relate to the pathophysiology of TGCT. Identifying biomarkers for early diagnosis and therapy response prediction for TGCT is critical. LncRNAs could be of clinical use in this cancer as well because of their strong tissue and disease specificity [240,241]. RNA sequencing and high-throughput gene chip technology offer dependable ways to discover efficient lncRNA biomarkers. Yang et al. found that the MEG3 modulates TGCT progression through the PTEN/PI3K/AKT pathway [242]. Another study discovered that the expression of LNC00467 was strongly connected to the pathological grade and poor prognosis of TGCT, and that it might facilitate TGCT cell invasion and migration via controlling the expression of AKT3 and influencing AKT phosphorylation [243]. The selection of a suitable treatment technique is currently a significant problem in the study of testicular cancer [244]. Studies have indicated that classifying cancer patients according to their clinical features, such as molecular markers, stage, and grade, and choosing the right therapy can enhance patient prognosis and lessen side effects from surgery, radiation, and chemotherapy [245]. To direct TGCT treatment, it is crucial to extract detailed medical information from large-scale databases such as TCGA, high-throughput GEO, epidemiological, and prognostic databases. Testis-specific lncRNA RFPL3S expression profiling was used for the first time by Guo et al. to discriminate seminoma from non-seminoma and prognostically predict TGCT [243]. Elevated RFPL3S expression predicted longer disease-free and progression-free intervals in TGCT patients. Furthermore, both genetic (copy number variation) and epigenetic (DNA methylation) factors influenced RFPL3S expression, a tumor suppressor that significantly reduced TGCT cell invasion and proliferation in vitro. An essential barrier to stop tumor spread in tissue is the ECM. The primary components, fibronectin and laminin, are coupled to integrin receptors on the cell membrane, which regulate cell shape, differentiation, and migration [246]. Through a number of signaling pathways, focal adhesion kinase is crucial for the control of the cell cycle, growth, adhesion, cytoskeletal assembly, motility, and survival [247]. Focal adhesion kinase is widely expressed in a variety of tumor types and plays a critical role in the development, invasion, and metastasis of malignancies. It may emerge as a new target for tumor treatment [248]. In addition to its link with tumor cell growth and migration, higher RFPL3S expression has been found in patients who benefit and respond to immunotherapy. RFPL3S has been linked to the PI3K/AKT/mTOR, b-catenin/Wnt, and Hippo pathways, all of which are linked to immunotherapy [249,250,251,252,253,254,255]. These findings suggest that RFPL3S may be a reliable biomarker for predicting immunotherapy efficacy in TGCT patients. Furthermore, the vast majority of patients, including those with metastatic disease, have favorable prognosis or recovery after treatment since seminoma cells are typically particularly susceptible to platinum-based pharmacological therapies. However, some cisplatin (CDDP)-resistant cases have also been documented, typically with poor prognosis [256]. Several mechanisms have been proposed to explain the high sensitivity or resistance to CDDP [257,258]. One of these proposed mechanisms was the TDRG1 gene, which regulates the activity of the PI3K/Akt signaling pathway to regulate CDDP sensitivity [259]. The H19/miRNA-106b-5p/TDRG1 axis was confirmed in a CDDP-resistant environment and in homeostatic seminoma [259]. The “sponge” function of lncRNA H19 caused miRNA-106b-5p to be sequestered, which promoted TDRG1 production. Furthermore, the H19/miR-NA-106b-5p/NDRG1 axis was recently identified as a potential target for the treatment of seminoma and CDDP-resistant cancers [260].

## 6. Conclusions

LncRNAs have been found to play important roles in a variety of biological processes, including the pathogenesis of many complex human diseases, including cancer. Their detailed regulation mechanisms in cancer initiation and progression largely remain unknown. Currently, functions of only a few lncRNA have been identified. Here, we have attempted to summarize recent advances in understanding the mechanisms and functions of lncRNAs in genitourinary cancers. Specifically, we focused on the roles of newly identified lncRNAs as oncogenes and tumor suppressors, as well as the molecular pathways in which they are involved. We have also discussed their potential utility as biomarkers for the cancer diagnosis and prognosis. In conclusion, lncRNAs, like miRNAs, play crucial roles in cancer progression by regulating gene regulatory networks. In fact, in clinical diagnosis and treatment, they have emerged as important new players. Despite the fact that a lot is known about their functions, substantial challenges still remain to be tackled. To fully elucidate how these ncRNAs regulate gene expression, we will have to further investigate their functional motifs, secondary or tertiary structures, as well as the development of advanced bioinformatics methods to predict the target genes. Moreover, a better understanding of their role in gene regulation will provide new insights into cancer diagnosis and therapy and help to develop novel therapeutic strategies. RNA-based therapies represent a promising field that could provide great advantages in the treatment of aggressive cancers. In genitourinary cancers, particularly in prostate and renal cancers, some progress has been made. However translation of novel therapeutic approaches into routine clinical practice is far from satisfactory. If the outstanding success of mRNA-based vaccines, both in terms of efficacy and remarkable adaptability, in the management of the current SARS-CoV-2 pandemic [261] is anything to go by, we can anticipate new impetus and hope for RNA based therapies in cancer [262].

## Figures and Tables

**Table 1:** Identified oncogenic role for lncRNAs in renal cell carcinoma.

LncRNA	Chr Location	Oncogenic Mechanisms	Specified Actions	Supposed Role	Expression Pattern *	Refs.
MALAT1	11q13	EMT	(i) Inhibition of ZEB2 suppression by ceRNA; (ii) EZH2-mediated epigenetic reprogramming.	Oncogene	UP	[12,14]
ZFAS1	20q13	Proliferation	(i) Control of miR-10a/SKA1 pathway; (ii) Control of miR-150-5P/HMGA2 molecular axis.	Oncogene	UP	[15]
CYTOR	2p11.2	Proliferation	(i) Control of miR-136-5-p/MAT2B/BAG3 axis; (ii) Control of miR-3679-5p/MACC1 axis.	Oncogene	UP	[16]
ROR	8q21.31	Proliferation	Control of miR-206/VEGF axis	Oncogene	UP	[24]
UCA1	9p13.12	Proliferation	Control of miR-182-5p/DLL4 axis	Oncogene	UP	[25]
H19	11p15	Proliferation	ceRNA preventing E2F1 repression	Oncogene	UP	[28,29]
HOTAIR	12q13	Proliferation Angiogenesis	(i) Action as a ceRNA preventing HIF1α repression; (ii) Epigenetic reprogramming by EZH2; (iii) IGFBP 2 expression’s upregulation.	Oncogene	UP	[31]
CADM1-AS1	11q23	Adhesion	Upregulation of CADM1 expression	Suppressor	DOWN	[36]
GAS5	1q25	Apoptosis	Promotion of P53-dependent and P53- independent apoptosis	Oncogene	UP	[37,38,39,40]

* UP: upregulation; DOWN: downregulation.

**Table 2:** Identified oncogenic role for lncRNAs in bladder cancer.

LncRNA	Chr Location	Oncogenic Mechanisms	Specified Actions	Supposed Role	Expression Pattern *	Refs.
MEG3	14q32	Apoptosis	(i) Reduction of MMP2 and MMP9 expression; (ii) Reduction of Bcl-2 expression	Suppressor	DOWN	[101]
UCA1	19p13	EMT Metabolism Cell cycle	(i) Induction of Wnt6 expression; (ii) CREB recruitment to enhance miRNA-196a-5p expression	Oncogene	UP	[105,106]
SLC16A1-AS1	1p13	Proliferation Metabolism	(i) Stimulation of glycolysis and mitochondrial respiration; (ii) Increased β-oxidation of fatty acids	Oncogene	UP	[76]
GAS6-AS2	13q34	Proliferation Cell cycle	CDK9 overexpression	Oncogene	UP	[80]
PVT1	8q24	Proliferation	CDK1 upregulation	Oncogene	UP	[83]
PTENP1	9p13	Apoptosis	(i) Sponge of miR-103a; (ii) Regulation of miR-103a/PDCD4 axis	Suppressor	DOWN	[93]
DLEU1	13q14	Proliferation	Increase of HS3ST3B1 expression by inhibiting miRNA-99b	Oncogene	UP	[102]
MST1P2	1p36	Increased chemoresistance	Enhancing of SIRT3 expression by inhibiting miRNA-133b	Oncogene	UP	[104]
H19	11p15	EMT Apoptosis	(i) β-catenin upregulation by EZH2 binding; (ii) Action as a ceRNA preventing DNMT3B degradation	Oncogene	UP	[107,108,109,110]
MALAT1	11q13	EMT	(i) Direct binding and activation of SUZ12; (ii) ZEB1, ZEB2 and SNAI2 upregulation	Oncogene	UP	[115]
XIST	Xq13	AR pathway	ceRNA preventing AR suppression by miR-124	Oncogene	UP	[123]
UC8+	1p36.22	Proliferation	Sponge for miR-596, increasing the MMP9 expression	Oncogene	UP	[120,121,122]

* UP: upregulation; DOWN: downregulation.

**Table 3:** Identified oncogenic role for lncRNAs in prostate cancer.

LncRNA	Chr Location	Oncogenic Mechanisms	Specified Actions	Supposed Role	Expression Pattern *	Refs.
ARLNC1	16q23	AR pathway	Stabilization of AR miRNA	Oncogene	UP	[153]
PRNCR1	8q24	AR pathway	Direct AR binding and activation	Oncogene	UP	[155]
PCGEM1	2q32	AR pathway Proliferation Invasion	(i) Direct AR binding and activation; (ii) Activation of MYC	Oncogene	UP	[154,156]
MALAT1	11q13	Proliferation Invasion	Downregulation of multiple tumor suppressor genes through EZH2 binding	Oncogene	UP	[198]
UCA1	19p13	Proliferation Invasion	Promotion of expression level of CXCR4 by sponging miR-204	Oncogene	UP	[187]
SChLAP1	2q31	MAPK signaling	(i) SWI/SNF transcriptional regulator complex activation; (ii) MAPK1 inhibitor miR-198 repression	Oncogene	UP	[164]
AFAP1-AS1	4p16	Proliferation	Regulation of miR-15b/IGF1R axis	Oncogene	UP	[162]
Linc00963	9q34.11	Proliferation Invasion	(i) By taking part in the transactivation of EGFR, prostate cancer promotion can change from an androgen-dependent mode to an androgen-independent mode; (ii) binding of EZH2 and suppression of p21 expression	Oncogene	UP	[166]
SNHG7	9q34.3	Proliferation Cell cycle	Regulation of miR-503 and cyclin D	Oncogene	UP	[168]
LOXL1-AS1	15q24	Proliferation Cell cycle	Expression modulation of miR-541-3p and of cyclin D	Oncogene	UP	[169]
PVT1	8q24	Apoptosis	KIF23 expression promotion by reducing miR-15a-5p expression	Oncogene	UP	[173]
ATB	14q11.2	Proliferation Invasion Cell cycle EMT	(i) Increase of cyclin E and cyclin D1 expression levels; (ii) Regulation of PI3K-AKT-mTOR and ERK signaling pathways	Oncogene	UP	[190]
ROR	18q21.31	Proliferation Invasion Migration	(i) ceRNA reducing miRNA-145; (ii) Regulation of PI3K\Akt pathway	Oncogene	UP	[200]

* UP: upregulation; DOWN: downregulation.

## Data Availability

Data sharing not applicable.

## References

[1] Zarrabi K., Paroya A., Wu S. (2019). Emerging therapeutic agents for genitourinary cancers. J. Hematol. Oncol..

[2] Ukleja J., Kusaka E., Miyamoto D.T. (2021). Immunotherapy Combined With Radiation Therapy for Genitourinary Malignancies. Front. Oncol..

[3] Galon J., Bruni D. (2019). Approaches to Treat Immune Hot, Altered and Cold Tumours with Combination Immunotherapies. Nat. Rev. Drug Discov..

[4] Park E.G., Pyo S.J., Cui Y., Yoon S.H., Nam J.W. (2022). Tumor immune microenvironment lncRNAs. Brief Bioinform..

[5] Siegel R.L., Miller K.D., Jemal A. (2018). Cancer Statistics, 2018. CA Cancer J. Clin..

[6] Perazella M.A., Dreicer R., Rosner M.H. (2018). Renal Cell Carcinoma for the Nephrologist. Kidney Int..

[7] Chen J., Chen Y., Gu L., Li X., Gao Y., Lyu X., Chen L., Luo G., Wang L., Xie Y. (2016). LncRNAs Act as Prognostic and Diagnostic Biomarkers in Renal Cell Carcinoma: A Systematic Review and Meta-Analysis. Oncotarget.

[8] Hanahan D., Weinberg R.A. (2000). The Hallmarks of Cancer. Cell.

[9] Hanahan D., Weinberg R.A. (2011). Hallmarks of Cancer: The next Generation. Cell.

[10] Wang J., Xu A.M., Zhang J.Y., He X.M., Pan Y.S., Cheng G., Qin C., Hua L.X., Wang Z.J. (2016). Prognostic Significance of Long Non-Coding RNA MALAT-1 in Various Human Carcinomas: A Meta-Analysis. Genet. Mol. Res..

[11] Zhang H., Yang F., Chen S.-J., Che J., Zheng J. (2015). Upregulation of Long Non-Coding RNA MALAT1 Correlates with Tumor Progression and Poor Prognosis in Clear Cell Renal Cell Carcinoma. Tumour Biol..

[12] Hirata H., Hinoda Y., Shahryari V., Deng G., Nakajima K., Tabatabai Z.L., Ishii N., Dahiya R. (2015). Long Noncoding RNA MALAT1 Promotes Aggressive Renal Cell Carcinoma through Ezh2 and Interacts with MiR-205. Cancer Res..

[13] Yang W., Lee D.Y., Ben-David Y. (2011). The Roles of MicroRNAs in Tumorigenesis and Angiogenesis. Int. J. Physiol. Pathophysiol. Pharm..

[14] Xiao H., Tang K., Liu P., Chen K., Hu J., Zeng J., Xiao W., Yu G., Yao W., Zhou H. (2015). LncRNA MALAT1 Functions as a Competing Endogenous RNA to Regulate ZEB2 Expression by Sponging MiR-200s in Clear Cell Kidney Carcinoma. Oncotarget.

[15] Dong D., Mu Z., Wei N., Sun M., Wang W., Xin N., Shao Y., Zhao C. (2019). Long Non-Coding RNA ZFAS1 Promotes Proliferation and Metastasis of Clear Cell Renal Cell Carcinoma via Targeting MiR-10a/SKA1 Pathway. Biomed. Pharm..

[16] Wang D., Zhu X., Siqin B., Ren C., Yi F. (2022). Long Non-Coding RNA CYTOR Modulates Cancer Progression through MiR-136-5p/MAT2B Axis in Renal Cell Carcinoma. Toxicol. Appl. Pharm..

[17] Wu T., Zhang D.-L., Wang J.-M., Jiang J.-Y., Du X., Zeng X.-Y., Du Z.-X. (2020). TRIM29 Inhibits MiR-873-5P Biogenesis via CYTOR to Upregulate Fibronectin 1 and Promotes Invasion of Papillary Thyroid Cancer Cells. Cell Death Dis..

[18] Zhu W., Wang J., Liu X., Xu Y., Zhai R., Zhang J., Wang M., Wang M., Liu L. (2022). LncRNA CYTOR Promotes Aberrant Glycolysis and Mitochondrial Respiration via HNRNPC-Mediated ZEB1 Stabilization in Oral Squamous Cell Carcinoma. Cell Death Dis..

[19] Liu Y., Geng X. (2022). Long Non-Coding RNA (LncRNA) CYTOR Promotes Hepatocellular Carcinoma Proliferation by Targeting the MicroRNA-125a-5p/LASP1 Axis. Bioengineered.

[20] Niu J., Li Z., Li F. (2019). Overexpressed MicroRNA-136 Works as a Cancer Suppressor in Gallbladder Cancer through Suppression of JNK Signaling Pathway via Inhibition of MAP2K4. Am. J. Physiol.-Gastrointest. Liver Physiol..

[21] Gao R.-Z., Que Q., Lin P., Pang Y.-Y., Wu H.-Y., Li X.-J., Chen G., He Y., Yang H. (2019). Clinical Roles of MiR-136-5p and Its Target Metadherin in Thyroid Carcinoma. Am. J. Transl. Res..

[22] Han C., Fu Y., Zeng N., Yin J., Li Q. (2020). LncRNA FAM83H-AS1 Promotes Triple-Negative Breast Cancer Progression by Regulating the MiR-136-5p/Metadherin Axis. Aging.

[23] Li M., Wang Q., Xue F., Wu Y. (2019). LncRNA-CYTOR Works as an Oncogene Through the CYTOR/MiR-3679-5p/MACC1 Axis in Colorectal Cancer. DNA Cell Biol..

[24] Shi J., Zhang D., Zhong Z., Zhang W. (2019). LncRNA ROR Promotes the Progression of Renal Cell Carcinoma through the MiR-206/VEGF Axis. Mol. Med. Rep..

[25] Wang W., Hu W., Wang Y., An Y., Song L., Shang P., Yue Z. (2020). Long Non-Coding RNA UCA1 Promotes Malignant Phenotypes of Renal Cancer Cells by Modulating the MiR-182-5p/DLL4 Axis as a CeRNA. Mol. Cancer.

[26] Zhang H., Li W., Gu W., Yan Y., Yao X., Zheng J. (2019). MALAT1 Accelerates the Development and Progression of Renal Cell Carcinoma by Decreasing the Expression of MiR-203 and Promoting the Expression of BIRC5. Cell Prolif..

[27] Wu W., Hu Q., Nie E., Yu T., Wu Y., Zhi T., Jiang K., Shen F., Wang Y., Zhang J. (2017). Hypoxia Induces H19 Expression through Direct and Indirect Hif-1α Activity, Promoting Oncogenic Effects in Glioblastoma. Sci. Rep..

[28] Wang L., Cai Y., Zhao X., Jia X., Zhang J., Liu J., Zhen H., Wang T., Tang X., Liu Y. (2015). Down-Regulated Long Non-Coding RNA H19 Inhibits Carcinogenesis of Renal Cell Carcinoma. Neoplasma.

[29] He H., Wang N., Yi X., Tang C., Wang D. (2017). Long Non-Coding RNA H19 Regulates E2F1 Expression by Competitively Sponging Endogenous MiR-29a-3p in Clear Cell Renal Cell Carcinoma. Cell Biosci..

[30] Raveh E., Matouk I.J., Gilon M., Hochberg A. (2015). The H19 Long Non-Coding RNA in Cancer Initiation, Progression and Metastasis—A Proposed Unifying Theory. Mol. Cancer.

[31] Wu Y., Liu J., Zheng Y., You L., Kuang D., Liu T. (2014). Suppressed Expression of Long Non-Coding RNA HOTAIR Inhibits Proliferation and Tumourigenicity of Renal Carcinoma Cells. Tumour Biol..

[32] Rinn J.L., Kertesz M., Wang J.K., Squazzo S.L., Xu X., Brugmann S.A., Goodnough L.H., Helms J.A., Farnham P.J., Segal E. (2007). Functional Demarcation of Active and Silent Chromatin Domains in Human HOX Loci by Noncoding RNAs. Cell.

[33] Katayama H., Tamai K., Shibuya R., Nakamura M., Mochizuki M., Yamaguchi K., Kawamura S., Tochigi T., Sato I., Okanishi T. (2017). Long Non-Coding RNA HOTAIR Promotes Cell Migration by Upregulating Insulin Growth Factor-Binding Protein 2 in Renal Cell Carcinoma. Sci. Rep..

[34] Hong Q., Li O., Zheng W., Xiao W.-Z., Zhang L., Wu D., Cai G.-Y., He J.C., Chen X.-M. (2017). LncRNA HOTAIR Regulates HIF-1α/AXL Signaling through Inhibition of MiR-217 in Renal Cell Carcinoma. Cell Death Dis..

[35] Ding J., Yeh C.-R., Sun Y., Lin C., Chou J., Ou Z., Chang C., Qi J., Yeh S. (2018). Estrogen Receptor β Promotes Renal Cell Carcinoma Progression via Regulating LncRNA HOTAIR-MiR-138/200c/204/217 Associated CeRNA Network. Oncogene.

[36] Yao J., Chen Y., Wang Y., Liu S., Yuan X., Pan F., Geng P. (2014). Decreased Expression of a Novel LncRNA CADM1-AS1 Is Associated with Poor Prognosis in Patients with Clear Cell Renal Cell Carcinomas. Int. J. Clin. Exp. Pathol..

[37] Zhang H.-M., Yang F.-Q., Yan Y., Che J.-P., Zheng J.-H. (2014). High Expression of Long Non-Coding RNA SPRY4-IT1 Predicts Poor Prognosis of Clear Cell Renal Cell Carcinoma. Int. J. Clin. Exp. Pathol..

[38] Song S., Wu Z., Wang C., Liu B., Ye X., Chen J., Yang Q., Ye H., Xu B., Wang L. (2014). RCCRT1 Is Correlated with Prognosis and Promotes Cell Migration and Invasion in Renal Cell Carcinoma. Urology.

[39] Xue S., Li Q.-W., Che J.-P., Guo Y., Yang F.-Q., Zheng J.-H. (2015). Decreased Expression of Long Non-Coding RNA NBAT-1 Is Associated with Poor Prognosis in Patients with Clear Cell Renal Cell Carcinoma. Int. J. Clin. Exp. Pathol..

[40] Su H., Sun T., Wang H., Shi G., Zhang H., Sun F., Ye D. (2017). Decreased TCL6 Expression Is Associated with Poor Prognosis in Patients with Clear Cell Renal Cell Carcinoma. Oncotarget.

[41] Xie Y.-H., Chen Y.-X., Fang J.-Y. (2020). Comprehensive Review of Targeted Therapy for Colorectal Cancer. Signal Transduct. Target. Ther..

[42] Friedmann Angeli J.P., Schneider M., Proneth B., Tyurina Y.Y., Tyurin V.A., Hammond V.J., Herbach N., Aichler M., Walch A., Eggenhofer E. (2014). Inactivation of the Ferroptosis Regulator Gpx4 Triggers Acute Renal Failure in Mice. Nat. Cell Biol..

[43] Yang W.S., Stockwell B.R. (2016). Ferroptosis: Death by Lipid Peroxidation. Trends Cell Biol..

[44] Stockwell B.R., Friedmann Angeli J.P., Bayir H., Bush A.I., Conrad M., Dixon S.J., Fulda S., Gascón S., Hatzios S.K., Kagan V.E. (2017). Ferroptosis: A Regulated Cell Death Nexus Linking Metabolism, Redox Biology, and Disease. Cell.

[45] Wu Z., Huang X., Cai M., Huang P. (2022). Potential Biomarkers for Predicting the Overall Survival Outcome of Kidney Renal Papillary Cell Carcinoma: An Analysis of Ferroptosis-Related LNCRNAs. BMC Urol..

[46] Song X., Long D. (2020). Nrf2 and Ferroptosis: A New Research Direction for Neurodegenerative Diseases. Front. Neurosci..

[47] Li C.H., Chen Y. (2013). Targeting Long Non-Coding RNAs in Cancers: Progress and Prospects. Int. J. Biochem. Cell Biol..

[48] Ling H., Fabbri M., Calin G.A. (2013). MicroRNAs and Other Non-Coding RNAs as Targets for Anticancer Drug Development. Nat. Rev. Drug Discov..

[49] Lennox K.A., Behlke M.A. (2016). Cellular Localization of Long Non-Coding RNAs Affects Silencing by RNAi More than by Antisense Oligonucleotides. Nucleic Acids Res..

[50] Bray F., Ferlay J., Soerjomataram I., Siegel R.L., Torre L.A., Jemal A. (2018). Global Cancer Statistics 2018: GLOBOCAN Estimates of Incidence and Mortality Worldwide for 36 Cancers in 185 Countries. CA Cancer J. Clin..

[51] Yu E.Y.-W., Zhang H., Fu Y., Chen Y.-T., Tang Q.-Y., Liu Y.-X., Zhang Y.-X., Wang S.-Z., Wesselius A., Li W.-C. (2022). Integrative Multi-Omics Analysis for the Determination of Non-Muscle Invasive vs. Muscle Invasive Bladder Cancer: A Pilot Study. Curr. Oncol..

[52] Cumberbatch M.G.K., Noon A.P., on behalf of the EAU Young Academic Urologists—Urothelial Cancer Working Party (2019). Epidemiology, Aetiology and Screening of Bladder Cancer. Transl. Androl. Urol..

[53] DeGeorge K.C., Holt H.R., Hodges S.C. (2017). Bladder Cancer: Diagnosis and Treatment. Am. Fam. Physician.

[54] Ashrafizadeh M., Hushmandi K., Hashemi M., Akbari M.E., Kubatka P., Raei M., Koklesova L., Shahinozzaman M., Mohammadinejad R., Najafi M. (2020). Role of MicroRNA/Epithelial-to-Mesenchymal Transition Axis in the Metastasis of Bladder Cancer. Biomolecules.

[55] Parizi P.K., Yarahmadi F., Tabar H.M., Hosseini Z., Sarli A., Kia N., Tafazoli A., Esmaeili S.-A. (2020). MicroRNAs and Target Molecules in Bladder Cancer. Med. Oncol..

[56] Böhmer D., Grün A. (2021). Lacking Evidence to Recommend Neoadjuvant Chemotherapy and Definitive Radiotherapy in Muscle-Invasive Bladder Cancer. Curr. Oncol. Rep..

[57] Witjes J.A., Bruins H.M., Cathomas R., Compérat E.M., Cowan N.C., Gakis G., Hernández V., Linares Espinós E., Lorch A., Neuzillet Y. (2021). European Association of Urology Guidelines on Muscle-Invasive and Metastatic Bladder Cancer: Summary of the 2020 Guidelines. Eur. Urol..

[58] Rachakonda P.S., Hosen I., de Verdier P.J., Fallah M., Heidenreich B., Ryk C., Wiklund N.P., Steineck G., Schadendorf D., Hemminki K. (2013). TERT Promoter Mutations in Bladder Cancer Affect Patient Survival and Disease Recurrence through Modification by a Common Polymorphism. Proc. Natl. Acad. Sci. USA.

[59] Allory Y., Beukers W., Sagrera A., Flández M., Marqués M., Márquez M., van der Keur K.A., Dyrskjot L., Lurkin I., Vermeij M. (2014). Telomerase Reverse Transcriptase Promoter Mutations in Bladder Cancer: High Frequency across Stages, Detection in Urine, and Lack of Association with Outcome. Eur. Urol..

[60] Kurtis B., Zhuge J., Ojaimi C., Ye F., Cai D., Zhang D., Fallon J.T., Zhong M. (2016). Recurrent TERT Promoter Mutations in Urothelial Carcinoma and Potential Clinical Applications. Ann. Diagn. Pathol..

[61] Leão R., Lee D., Figueiredo A., Hermanns T., Wild P., Komosa M., Lau I., Mistry M., Nunes N.M., Price A.J. (2019). Combined Genetic and Epigenetic Alterations of the TERT Promoter Affect Clinical and Biological Behavior of Bladder Cancer. Int. J. Cancer.

[62] Li Y., Qiao L., Zang Y., Ni W., Xu Z. (2020). Circular RNA FOXO3 Suppresses Bladder Cancer Progression and Metastasis by Regulating MiR-9-5p/TGFBR2. Cancer Manag. Res..

[63] Liu Q., Zhou Q., Zhong P. (2020). Circ_0067934 Increases Bladder Cancer Cell Proliferation, Migration and Invasion through Suppressing MiR-1304 Expression and Increasing Myc Expression Levels. Exp. Ther. Med..

[64] Wu S., Deng H., He H., Xu R., Wang Y., Zhu X., Zhang J., Zeng Q., Zhao X. (2020). The Circ_0004463/MiR-380-3p/FOXO1 Axis Modulates Mitochondrial Respiration and Bladder Cancer Cell Apoptosis. Cell Cycle.

[65] Du L., Zhang L., Sun F. (2022). Puerarin Inhibits the Progression of Bladder Cancer by Regulating Circ_0020394/MiR-328-3p/NRBP1 Axis. Cancer Biother. Radiopharm..

[66] Mojarrad M., Moghbeli M. (2020). Genetic and Molecular Biology of Bladder Cancer among Iranian Patients. Mol. Genet. Genom. Med..

[67] Huang W., Lu Y., Wang F., Huang X., Yu Z. (2020). Circular RNA CircRNA_103809 Accelerates Bladder Cancer Progression and Enhances Chemo-Resistance by Activation of MiR-516a-5p/FBXL18 Axis. Cancer Manag. Res..

[68] Ashrafizaveh S., Ashrafizadeh M., Zarrabi A., Husmandi K., Zabolian A., Shahinozzaman M., Aref A.R., Hamblin M.R., Nabavi N., Crea F. (2021). Long Non-Coding RNAs in the Doxorubicin Resistance of Cancer Cells. Cancer Lett..

[69] Mirzaei S., Zarrabi A., Hashemi F., Zabolian A., Saleki H., Ranjbar A., Seyed Saleh S.H., Bagherian M., Sharifzadeh S.O., Hushmandi K. (2021). Regulation of Nuclear Factor-KappaB (NF-ΚB) Signaling Pathway by Non-Coding RNAs in Cancer: Inhibiting or Promoting Carcinogenesis?. Cancer Lett..

[70] Wang W., Liu Z., Zhang X., Liu J., Gui J., Cui M., Li Y. (2020). MiR-211-5p Is down-Regulated and a Prognostic Marker in Bladder Cancer. J. Gene Med..

[71] Chen L., Yang X., Zhao J., Xiong M., Almaraihah R., Chen Z., Hou T. (2020). Circ_0008532 Promotes Bladder Cancer Progression by Regulation of the MiR-155-5p/MiR-330-5p/MTGR1 Axis. J. Exp. Clin. Cancer Res..

[72] Zhang C., Wang W., Lin J., Xiao J., Tian Y. (2019). LncRNA CCAT1 Promotes Bladder Cancer Cell Proliferation, Migration and Invasion. Int. Braz. J. Urol..

[73] Petrella G., Ciufolini G., Vago R., Cicero D.O. (2020). The Interplay between Oxidative Phosphorylation and Glycolysis as a Potential Marker of Bladder Cancer Progression. Int. J. Mol. Sci..

[74] Wang J.-Z., Zhu W., Han J., Yang X., Zhou R., Lu H.-C., Yu H., Yuan W.-B., Li P.-C., Tao J. (2021). The Role of the HIF-1α/ALYREF/PKM2 Axis in Glycolysis and Tumorigenesis of Bladder Cancer. Cancer Commun..

[75] Mamouni K., Kim J., Lokeshwar B.L., Kallifatidis G. (2021). ARRB1 Regulates Metabolic Reprogramming to Promote Glycolysis in Stem Cell-Like Bladder Cancer Cells. Cancers.

[76] Logotheti S., Marquardt S., Gupta S.K., Richter C., Edelhäuser B.A.H., Engelmann D., Brenmoehl J., Söhnchen C., Murr N., Alpers M. (2020). LncRNA-SLC16A1-AS1 Induces Metabolic Reprogramming during Bladder Cancer Progression as Target and Co-Activator of E2F1. Theranostics.

[77] Zhong X., Long Z., Wu S., Xiao M., Hu W. (2018). LncRNA-SNHG7 Regulates Proliferation, Apoptosis and Invasion of Bladder Cancer Cells Assurance Guidelines. J. BUON.

[78] Wu X., Yan T., Wang Z., Wu X., Cao G., Zhang C. (2017). LncRNA ZEB2-AS1 Promotes Bladder Cancer Cell Proliferation and Inhibits Apoptosis by Regulating MiR-27b. Biomed. Pharm..

[79] Liu X., Song J., Zhang Y., Wang H., Sun H., Feng X., Hou M., Chen G., Tang Q., Ji M. (2020). ASF1B Promotes Cervical Cancer Progression through Stabilization of CDK9. Cell Death Dis..

[80] Rui X., Wang L., Pan H., Gu T., Shao S., Leng J. (2019). LncRNA GAS6-AS2 Promotes Bladder Cancer Proliferation and Metastasis via GAS6-AS2/MiR-298/CDK9 Axis. J. Cell. Mol. Med..

[81] Jiang W., Zhu D., Wang C., Zhu Y. (2021). Tumor Suppressing Effects of Tristetraprolin and Its Small Double-Stranded RNAs in Bladder Cancer. Cancer Med..

[82] Yuan X., Li T., Xiao E., Zhao H., Li Y., Fu S., Gan L., Wang Z., Zheng Q., Wang Z. (2014). Licochalcone B Inhibits Growth of Bladder Cancer Cells by Arresting Cell Cycle Progression and Inducing Apoptosis. Food Chem. Toxicol..

[83] Tian Z., Cao S., Li C., Xu M., Wei H., Yang H., Sun Q., Ren Q., Zhang L. (2019). LncRNA PVT1 Regulates Growth, Migration, and Invasion of Bladder Cancer by MiR-31/CDK1. J. Cell. Physiol..

[84] Qiao Z., Dai H., Zhang Y., Li Q., Zhao M., Yue T. (2020). LncRNA NCK1-AS1 Promotes Cancer Cell Proliferation and Increase Cell Stemness in Urinary Bladder Cancer Patients by Downregulating MiR-143. Cancer Manag. Res..

[85] Wee I., Syn N., Sethi G., Goh B.C., Wang L. (2019). Role of Tumor-Derived Exosomes in Cancer Metastasis. Biochim. Biophys. Acta Rev. Cancer.

[86] Dai J., Su Y., Zhong S., Cong L., Liu B., Yang J., Tao Y., He Z., Chen C., Jiang Y. (2020). Exosomes: Key Players in Cancer and Potential Therapeutic Strategy. Signal Transduct. Target. Ther..

[87] Van den Boorn J.G., Dassler J., Coch C., Schlee M., Hartmann G. (2013). Exosomes as Nucleic Acid Nanocarriers. Adv. Drug Deliv. Rev..

[88] Schey K.L., Luther J.M., Rose K.L. (2015). Proteomics Characterization of Exosome Cargo. Methods.

[89] Skotland T., Sandvig K., Llorente A. (2017). Lipids in Exosomes: Current Knowledge and the Way Forward. Prog. Lipid Res..

[90] Puhka M., Takatalo M., Nordberg M.-E., Valkonen S., Nandania J., Aatonen M., Yliperttula M., Laitinen S., Velagapudi V., Mirtti T. (2017). Metabolomic Profiling of Extracellular Vesicles and Alternative Normalization Methods Reveal Enriched Metabolites and Strategies to Study Prostate Cancer-Related Changes. Theranostics.

[91] Weng J., Xiang X., Ding L., Wong A.L.-A., Zeng Q., Sethi G., Wang L., Lee S.C., Goh B.C. (2021). Extracellular Vesicles, the Cornerstone of next-Generation Cancer Diagnosis?. Semin. Cancer Biol..

[92] Kok V.C., Yu C.-C. (2020). Cancer-Derived Exosomes: Their Role in Cancer Biology and Biomarker Development. Int. J. Nanomed..

[93] Zheng R., Du M., Wang X., Xu W., Liang J., Wang W., Lv Q., Qin C., Chu H., Wang M. (2018). Exosome–Transmitted Long Non-Coding RNA PTENP1 Suppresses Bladder Cancer Progression. Mol. Cancer.

[94] Yu W.-D., Wang H., He Q.-F., Xu Y., Wang X.-C. (2018). Long Noncoding RNAs in Cancer-Immunity Cycle. J. Cell. Physiol..

[95] Abildgaard C., Do Canto L.M., Steffensen K.D., Rogatto S.R. (2019). Long Non-Coding RNAs Involved in Resistance to Chemotherapy in Ovarian Cancer. Front. Oncol..

[96] Prabhu K.S., Raza A., Karedath T., Raza S.S., Fathima H., Ahmed E.I., Kuttikrishnan S., Therachiyil L., Kulinski M., Dermime S. (2020). Non-Coding RNAs as Regulators and Markers for Targeting of Breast Cancer and Cancer Stem Cells. Cancers.

[97] Zhang L., Li L., Zhan Y., Wang J., Zhu Z., Zhang X. (2020). Identification of Immune-Related LncRNA Signature to Predict Prognosis and Immunotherapeutic Efficiency in Bladder Cancer. Front. Oncol..

[98] Liu S., Chen L., Zhao H., Li Q., Hu R., Wang H. (2020). Integrin Β8 Facilitates Tumor Growth and Drug Resistance through a Y-Box Binding Protein 1-Dependent Signaling Pathway in Bladder Cancer. Cancer Sci..

[99] Huang Z., Wang T., Xia W., Li Q., Chen X., Liu X., Wei P., Xu W., Lv M. (2020). Oblongifolin C Reverses GEM Resistance via Suppressing Autophagy Flux in Bladder Cancer Cells. Exp. Ther. Med..

[100] Xiong Y., Ju L., Yuan L., Chen L., Wang G., Xu H., Peng T., Luo Y., Xiao Y., Wang X. (2021). KNSTRN Promotes Tumorigenesis and Gemcitabine Resistance by Activating AKT in Bladder Cancer. Oncogene.

[101] Feng S.Q., Zhang X.Y., Fan H.T., Sun Q.J., Zhang M. (2018). Up-Regulation of LncRNA MEG3 Inhibits Cell Migration and Invasion and Enhances Cisplatin Chemosensitivity in Bladder Cancer Cells. Neoplasma.

[102] Li Y., Shi B., Dong F., Zhu X., Liu B., Liu Y. (2019). Long Non-Coding RNA DLEU1 Promotes Cell Proliferation, Invasion, and Confers Cisplatin Resistance in Bladder Cancer by Regulating the MiR-99b/HS3ST3B1 Axis. Front. Genet..

[103] Li L., Zeng H., He X., Chen J. (2021). Sirtuin 3 Alleviates Diabetic Cardiomyopathy by Regulating TIGAR and Cardiomyocyte Metabolism. J. Am. Heart Assoc..

[104] Chen J., Li Y., Li Z., Cao L. (2020). LncRNA MST1P2/MiR-133b Axis Affects the Chemoresistance of Bladder Cancer to Cisplatin-Based Therapy via Sirt1/P53 Signaling. J. Biochem. Mol. Toxicol..

[105] Fan Y., Shen B., Tan M., Mu X., Qin Y., Zhang F., Liu Y. (2014). Long Non-Coding RNA UCA1 Increases Chemoresistance of Bladder Cancer Cells by Regulating Wnt Signaling. FEBS J..

[106] Pan J., Li X., Wu W., Xue M., Hou H., Zhai W., Chen W. (2016). Long Non-Coding RNA UCA1 Promotes Cisplatin/Gemcitabine Resistance through CREB Modulating MiR-196a-5p in Bladder Cancer Cells. Cancer Lett..

[107] Elkin M., Shevelev A., Schulze E., Tykocinsky M., Cooper M., Ariel I., Pode D., Kopf E., de Groot N., Hochberg A. (1995). The Expression of the Imprinted H19 and IGF-2 Genes in Human Bladder Carcinoma. FEBS Lett..

[108] Ohana P., Kopf E., Bibi O., Ayesh S., Schneider T., Laster M., Tykocinski M., de Groot N., Hochberg A. (1999). The Expression of the H19 Gene and Its Function in Human Bladder Carcinoma Cell Lines. FEBS Lett..

[109] Ariel I., Sughayer M., Fellig Y., Pizov G., Ayesh S., Podeh D., Libdeh B.A., Levy C., Birman T., Tykocinski M.L. (2000). The Imprinted H19 Gene Is a Marker of Early Recurrence in Human Bladder Carcinoma. Mol. Pathol..

[110] Luo M., Li Z., Wang W., Zeng Y., Liu Z., Qiu J. (2013). Long Non-Coding RNA H19 Increases Bladder Cancer Metastasis by Associating with EZH2 and Inhibiting E-Cadherin Expression. Cancer Lett..

[111] Chen L.-H., Hsu W.-L., Tseng Y.-J., Liu D.-W., Weng C.-F. (2016). Involvement of DNMT 3B Promotes Epithelial-Mesenchymal Transition and Gene Expression Profile of Invasive Head and Neck Squamous Cell Carcinomas Cell Lines. BMC Cancer.

[112] Li S., Yu Z., Chen S.-S., Li F., Lei C.-Y., Chen X.-X., Bao J.-M., Luo Y., Lin G.-Z., Pang S.-Y. (2015). The YAP1 Oncogene Contributes to Bladder Cancer Cell Proliferation and Migration by Regulating the H19 Long Noncoding RNA. Urol. Oncol..

[113] Han Y., Liu Y., Nie L., Gui Y., Cai Z. (2013). Inducing Cell Proliferation Inhibition, Apoptosis, and Motility Reduction by Silencing Long Noncoding Ribonucleic Acid Metastasis-Associated Lung Adenocarcinoma Transcript 1 in Urothelial Carcinoma of the Bladder. Urology.

[114] Wang D., Ding L., Wang L., Zhao Y., Sun Z., Karnes R.J., Zhang J., Huang H. (2015). LncRNA MALAT1 Enhances Oncogenic Activities of EZH2 in Castration-Resistant Prostate Cancer. Oncotarget.

[115] Fan Y., Shen B., Tan M., Mu X., Qin Y., Zhang F., Liu Y. (2014). TGF-β-Induced Upregulation of Malat1 Promotes Bladder Cancer Metastasis by Associating with Suz12. Clin. Cancer Res..

[116] Ying L., Chen Q., Wang Y., Zhou Z., Huang Y., Qiu F. (2012). Upregulated MALAT-1 Contributes to Bladder Cancer Cell Migration by Inducing Epithelial-to-Mesenchymal Transition. Mol. Biosyst..

[117] Zhao Y.-H., Liu Y.-L., Fei K.-L., Li P. (2020). Long Non-Coding RNA HOTAIR Modulates the Progression of Preeclampsia through Inhibiting MiR-106 in an EZH2-Dependent Manner. Life Sci..

[118] Ashrafizadeh M., Gholami M.H., Mirzaei S., Zabolian A., Haddadi A., Farahani M.V., Kashani S.H., Hushmandi K., Najafi M., Zarrabi A. (2021). Dual Relationship between Long Non-Coding RNAs and STAT3 Signaling in Different Cancers: New Insight to Proliferation and Metastasis. Life Sci..

[119] Terreri S., Durso M., Colonna V., Romanelli A., Terracciano D., Ferro M., Perdonà S., Castaldo L., Febbraio F., de Nigris F. (2016). New Cross-Talk Layer between Ultraconserved Non-Coding RNAs, MicroRNAs and Polycomb Protein YY1 in Bladder Cancer. Genes.

[120] Olivieri M., Ferro M., Terreri S., Durso M., Romanelli A., Avitabile C., De Cobelli O., Messere A., Bruzzese D., Vannini I. (2016). Long Non-Coding RNA Containing Ultraconserved Genomic Region 8 Promotes Bladder Cancer Tumorigenesis. Oncotarget.

[121] Liu X., Zou H., Slaughter C., Wang X. (1997). DFF, a Heterodimeric Protein That Functions Downstream of Caspase-3 to Trigger DNA Fragmentation during Apoptosis. Cell.

[122] Terreri S., Mancinelli S., Ferro M., Vitale M.C., Perdonà S., Castaldo L., Gigantino V., Mercadante V., Cecio R.D., Aquino G. (2021). Subcellular Localization of Uc.8+ as a Prognostic Biomarker in Bladder Cancer Tissue. Cancers.

[123] Xiong Y., Wang L., Li Y., Chen M., He W., Qi L. (2017). The Long Non-Coding RNA XIST Interacted with MiR-124 to Modulate Bladder Cancer Growth, Invasion and Migration by Targeting Androgen Receptor (AR). Cell. Physiol. Biochem..

[124] Mirzaei S., Paskeh M.D.A., Hashemi F., Zabolian A., Hashemi M., Entezari M., Tabari T., Ashrafizadeh M., Raee P., Aghamiri S. (2022). Long Non-Coding RNAs as New Players in Bladder Cancer: Lessons from Pre-Clinical and Clinical Studies. Life Sci..

[125] Montazeri K., Montazeri J. (2020). Erdafitinib for the treatment of metastatic bladder cancer. Expert Rev Clin Pharm..

[126] Vafaei S., Zekiy A.O., Khanamir R.A., Zaman B.A., Ghayourvahdat A., Azimizonuzi H., Zamani M. (2022). Combination therapy with immune checkpoint inhibitors (ICIs); a new frontier. Cancer Cell Int..

[127] Wang L., Lu B., He M., Wang Y., Wang Z., Du L. (2022). Prostate Cancer Incidence and Mortality: Global Status and Temporal Trends in 89 Countries From 2000 to 2019. Front. Public Health.

[128] Wilt T.J., Jones K.M., Barry M.J., Andriole G.L., Culkin D., Wheeler T., Aronson W.J., Brawer M.K. (2017). Follow-up of Prostatectomy versus Observation for Early Prostate Cancer. N. Engl. J. Med..

[129] Loeb S., Folkvaljon Y., Makarov D.V., Bratt O., Bill-Axelson A., Stattin P. (2015). Five-Year Nationwide Follow-up Study of Active Surveillance for Prostate Cancer. Eur. Urol..

[130] Klotz L., Vesprini D., Sethukavalan P., Jethava V., Zhang L., Jain S., Yamamoto T., Mamedov A., Loblaw A. (2015). Long-Term Follow-Up of a Large Active Surveillance Cohort of Patients With Prostate Cancer. J. Clin. Oncol..

[131] Gillessen S., Omlin A., Attard G., de Bono J.S., Efstathiou E., Fizazi K., Halabi S., Nelson P.S., Sartor O., Smith M.R. (2015). Management of Patients with Advanced Prostate Cancer: Recommendations of the St Gallen Advanced Prostate Cancer Consensus Conference (APCCC) 2015. Ann. Oncol..

[132] Chen C.D., Welsbie D.S., Tran C., Baek S.H., Chen R., Vessella R., Rosenfeld M.G., Sawyers C.L. (2004). Molecular Determinants of Resistance to Antiandrogen Therapy. Nat. Med..

[133] Watson P.A., Arora V.K., Sawyers C.L. (2015). Emerging Mechanisms of Resistance to Androgen Receptor Inhibitors in Prostate Cancer. Nat. Rev. Cancer.

[134] Cornford P., Bellmunt J., Bolla M., Briers E., De Santis M., Gross T., Henry A.M., Joniau S., Lam T.B., Mason M.D. (2017). EAU-ESTRO-SIOG Guidelines on Prostate Cancer. Part II: Treatment of Relapsing, Metastatic, and Castration-Resistant Prostate Cancer. Eur. Urol..

[135] Eisenhauer E.A., Therasse P., Bogaerts J., Schwartz L.H., Sargent D., Ford R., Dancey J., Arbuck S., Gwyther S., Mooney M. (2009). New Response Evaluation Criteria in Solid Tumours: Revised RECIST Guideline (Version 1.1). Eur. J. Cancer.

[136] Kirby M., Hirst C., Crawford E.D. (2011). Characterising the Castration-Resistant Prostate Cancer Population: A Systematic Review. Int. J. Clin. Pract..

[137] de Bono J.S., Oudard S., Ozguroglu M., Hansen S., Machiels J.-P., Kocak I., Gravis G., Bodrogi I., Mackenzie M.J., Shen L. (2010). Prednisone plus Cabazitaxel or Mitoxantrone for Metastatic Castration-Resistant Prostate Cancer Progressing after Docetaxel Treatment: A Randomised Open-Label Trial. Lancet.

[138] Kantoff P.W., Higano C.S., Shore N.D., Berger E.R., Small E.J., Penson D.F., Redfern C.H., Ferrari A.C., Dreicer R., Sims R.B. (2010). Sipuleucel-T Immunotherapy for Castration-Resistant Prostate Cancer. N. Engl. J. Med..

[139] Fizazi K., Scher H.I., Molina A., Logothetis C.J., Chi K.N., Jones R.J., Staffurth J.N., North S., Vogelzang N.J., Saad F. (2012). Abiraterone Acetate for Treatment of Metastatic Castration-Resistant Prostate Cancer: Final Overall Survival Analysis of the COU-AA-301 Randomised, Double-Blind, Placebo-Controlled Phase 3 Study. Lancet Oncol..

[140] Scher H.I., Fizazi K., Saad F., Taplin M.-E., Sternberg C.N., Miller K., de Wit R., Mulders P., Chi K.N., Shore N.D. (2012). Increased Survival with Enzalutamide in Prostate Cancer after Chemotherapy. N. Engl. J. Med..

[141] Parker C., Nilsson S., Heinrich D., Helle S.I., O’Sullivan J.M., Fosså S.D., Chodacki A., Wiechno P., Logue J., Seke M. (2013). Alpha Emitter Radium-223 and Survival in Metastatic Prostate Cancer. N. Engl. J. Med..

[142] Ryan C.J., Smith M.R., Fizazi K., Saad F., Mulders P.F.A., Sternberg C.N., Miller K., Logothetis C.J., Shore N.D., Small E.J. (2015). Abiraterone Acetate plus Prednisone versus Placebo plus Prednisone in Chemotherapy-Naive Men with Metastatic Castration-Resistant Prostate Cancer (COU-AA-302): Final Overall Survival Analysis of a Randomised, Double-Blind, Placebo-Controlled Phase 3 Study. Lancet Oncol..

[143] Beer T.M., Armstrong A.J., Rathkopf D., Loriot Y., Sternberg C.N., Higano C.S., Iversen P., Evans C.P., Kim C.-S., Kimura G. (2017). Enzalutamide in Men with Chemotherapy-Naïve Metastatic Castration-Resistant Prostate Cancer: Extended Analysis of the Phase 3 PREVAIL Study. Eur. Urol..

[144] Scher H.I., Sawyers C.L. (2005). Biology of Progressive, Castration-Resistant Prostate Cancer: Directed Therapies Targeting the Androgen-Receptor Signaling Axis. J. Clin. Oncol..

[145] Hoang D.T., Iczkowski K.A., Kilari D., See W., Nevalainen M.T. (2016). Androgen Receptor-Dependent and -Independent Mechanisms Driving Prostate Cancer Progression: Opportunities for Therapeutic Targeting from Multiple Angles. Oncotarget.

[146] Wang X., Qi M., Zhang J., Sun X., Guo H., Pang Y., Zhang Q., Chen X., Zhang R., Liu Z. (2019). Differential Response to Neoadjuvant Hormonal Therapy in Prostate Cancer: Predictive Morphological Parameters and Molecular Markers. Prostate.

[147] De Winter J.A.R., Trapman J., Brinkmann A.O., Boersma W.J.A., Mulder E., Schroeder F.H., Claassen E., Van Der Kwast T.H. (1990). Androgen Receptor Heterogeneity in Human Prostatic Carcinomas Visualized by Immunohistochemistry. J. Pathol..

[148] Choucair K., Ejdelman J., Brimo F., Aprikian A., Chevalier S., Lapointe J. (2012). PTENgenomic Deletion Predicts Prostate Cancer Recurrence and Is Associated with Low AR Expression and Transcriptional Activity. BMC Cancer.

[149] Kojima S., Goto Y., Naya Y. (2017). The Roles of MicroRNAs in the Progression of Castration-Resistant Prostate Cancer. J. Hum. Genet..

[150] Shih J.-W., Wang L.-Y., Hung C.-L., Kung H.-J., Hsieh C.-L. (2015). Non-Coding RNAs in Castration-Resistant Prostate Cancer: Regulation of Androgen Receptor Signaling and Cancer Metabolism. Int. J. Mol. Sci..

[151] Ding L., Wang R., Shen D., Cheng S., Wang H., Lu Z., Zheng Q., Wang L., Xia L., Li G. (2021). Role of Noncoding RNA in Drug Resistance of Prostate Cancer. Cell Death Dis..

[152] Ramnarine V.R., Kobelev M., Gibb E.A., Nouri M., Lin D., Wang Y., Buttyan R., Davicioni E., Zoubeidi A., Collins C.C. (2019). The Evolution of Long Noncoding RNA Acceptance in Prostate Cancer Initiation, Progression, and Its Clinical Utility in Disease Management. Eur. Urol..

[153] Zhang Y., Pitchiaya S., Cieślik M., Niknafs Y.S., Tien J.C.-Y., Hosono Y., Iyer M.K., Yazdani S., Subramaniam S., Shukla S.K. (2018). Analysis of the Androgen Receptor–Regulated LncRNA Landscape Identifies a Role for ARLNC1 in Prostate Cancer Progression. Nat. Genet..

[154] Yang L., Lin C., Jin C., Yang J.C., Tanasa B., Li W., Merkurjev D., Ohgi K.A., Meng D., Zhang J. (2013). LncRNA-Dependent Mechanisms of Androgen-Receptor-Regulated Gene Activation Programs. Nature.

[155] Bardhan A., Banerjee A., Basu K., Pal D.K., Ghosh A. (2022). PRNCR1: A Long Non-Coding RNA with a Pivotal Oncogenic Role in Cancer. Hum. Genet..

[156] Aird J., Baird A.-M., Lim M.C.J., McDermott R., Finn S.P., Gray S.G. (2018). Carcinogenesis in Prostate Cancer: The Role of Long Non-Coding RNAs. Non-Coding RNA Res..

[157] Tam C., Wong J.H., Tsui S.K.W., Zuo T., Chan T.F., Ng T.B. (2019). LncRNAs with MiRNAs in Regulation of Gastric, Liver, and Colorectal Cancers: Updates in Recent Years. Appl. Microbiol. Biotechnol..

[158] Sun B., Liu C., Li H., Zhang L., Luo G., Liang S., Lü M. (2020). Research Progress on the Interactions between Long Non-coding RNAs and MicroRNAs in Human Cancer (Review). Oncol. Lett..

[159] Zhang J., Xu T., Liu L., Zhang W., Zhao C., Li S., Li J., Rao N., Le T.D. (2020). LMSM: A Modular Approach for Identifying LncRNA Related MiRNA Sponge Modules in Breast Cancer. PLoS Comput. Biol..

[160] López-Urrutia E., Bustamante Montes L.P., Ladrón de Guevara Cervantes D., Pérez-Plasencia C., Campos-Parra A.D. (2019). Crosstalk Between Long Non-Coding RNAs, Micro-RNAs and MRNAs: Deciphering Molecular Mechanisms of Master Regulators in Cancer. Front. Oncol..

[161] Hu C.-Y., Wu K.-Y., Lin T.-Y., Chen C.-C. (2022). The Crosstalk of Long Non-Coding RNA and MicroRNA in Castration-Resistant and Neuroendocrine Prostate Cancer: Their Interaction and Clinical Importance. Int. J. Mol. Sci..

[162] Liu B., Jiang H.-Y., Yuan T., Zhou W.-D., Xiang Z.-D., Jiang Q.-Q., Wu D.-L. (2021). Long Non-Coding RNA AFAP1-AS1 Facilitates Prostate Cancer Progression by Regulating MiR-15b/IGF1R Axis. Curr. Pharm. Des..

[163] Huang S., Liao Q., Li W., Deng G., Jia M., Fang Q., Ji H., Meng M. (2021). The LncRNA PTTG3P Promotes the Progression of CRPC via Upregulating PTTG1. Bull. Cancer.

[164] Li Y., Luo H., Xiao N., Duan J., Wang Z., Wang S. (2018). Long Noncoding RNA SChLAP1 Accelerates the Proliferation and Metastasis of Prostate Cancer via Targeting MiR-198 and Promoting the MAPK1 Pathway. Oncol. Res. Featur. Preclin. Clin. Cancer Ther..

[165] Wang L., Han S., Jin G., Zhou X., Li M., Ying X., Wang L., Wu H., Zhu Q. (2014). Linc00963: A Novel, Long Non-Coding RNA Involved in the Transition of Prostate Cancer from Androgen-Dependence to Androgen-Independence. Int. J. Oncol..

[166] Bai M., He C., Shi S., Wang M., Ma J., Yang P., Dong Y., Mou X., Han S. (2021). Linc00963 Promote Cell Proliferation and Tumor Growth in Castration-Resistant Prostate Cancer by Modulating MiR-655/TRIM24 Axis. Front. Oncol..

[167] Groner A.C., Cato L., de Tribolet-Hardy J., Bernasocchi T., Janouskova H., Melchers D., Houtman R., Cato A.C.B., Tschopp P., Gu L. (2016). TRIM24 Is an Oncogenic Transcriptional Activator in Prostate Cancer. Cancer Cell.

[168] Qi H., Wen B., Wu Q., Cheng W., Lou J., Wei J., Huang J., Yao X., Weng G. (2018). Long Noncoding RNA SNHG7 Accelerates Prostate Cancer Proliferation and Cycle Progression through Cyclin D1 by Sponging MiR-503. Biomed. Pharm..

[169] Long B., Li N., Xu X.-X., Li X.-X., Xu X.-J., Liu J.-Y., Wu Z.-H. (2018). Long Noncoding RNA LOXL1-AS1 Regulates Prostate Cancer Cell Proliferation and Cell Cycle Progression through MiR-541-3p and CCND1. Biochem. Biophys. Res. Commun..

[170] Zhao S., Zhang Y., Zhang Q., Wang F., Zhang D. (2014). Toll-like Receptors and Prostate Cancer. Front. Immunol..

[171] Galli R., Starace D., Busà R., Angelini D.F., Paone A., De Cesaris P., Filippini A., Sette C., Battistini L., Ziparo E. (2010). TLR Stimulation of Prostate Tumor Cells Induces Chemokine-Mediated Recruitment of Specific Immune Cell Types. J. Immunol..

[172] Sun M., Geng D., Li S., Chen Z., Zhao W. (2018). LncRNA PART1 Modulates Toll-like Receptor Pathways to Influence Cell Proliferation and Apoptosis in Prostate Cancer Cells. Biol. Chem..

[173] Wu H., Tian X., Zhu C. (2020). Knockdown of LncRNA PVT1 Inhibits Prostate Cancer Progression In Vitro and in Vivo by the Suppression of KIF23 through Stimulating MiR-15a-5p. Cancer Cell Int..

[174] Akoto T., Saini S. (2021). Role of Exosomes in Prostate Cancer Metastasis. Int. J. Mol. Sci..

[175] Keller E.T., Brown J. (2004). Prostate Cancer Bone Metastases Promote Both Osteolytic and Osteoblastic Activity. J. Cell. Biochem..

[176] Kretschmer A., Tilki D. (2017). Biomarkers in Prostate Cancer—Current Clinical Utility and Future Perspectives. Crit. Rev. Oncol. Hematol.

[177] Saini S. (2016). PSA and beyond: Alternative Prostate Cancer Biomarkers. Cell. Oncol..

[178] Bandyopadhyay S., Pai S.K., Gross S.C., Hirota S., Hosobe S., Miura K., Saito K., Commes T., Hayashi S., Watabe M. (2003). The Drg-1 Gene Suppresses Tumor Metastasis in Prostate Cancer. Cancer Res..

[179] Lingadahalli S., Jadhao S., Sung Y.Y., Chen M., Hu L., Chen X., Cheung E. (2018). Novel LncRNA LINC00844 Regulates Prostate Cancer Cell Migration and Invasion through AR Signaling. Mol. Cancer Res..

[180] Shanmugam M.K., Ahn K.S., Lee J.H., Kannaiyan R., Mustafa N., Manu K.A., Siveen K.S., Sethi G., Chng W.J., Kumar A.P. (2018). Celastrol Attenuates the Invasion and Migration and Augments the Anticancer Effects of Bortezomib in a Xenograft Mouse Model of Multiple Myeloma. Front. Pharm..

[181] Shanmugam M.K., Ahn K.S., Hsu A., Woo C.C., Yuan Y., Tan K.H.B., Chinnathambi A., Alahmadi T.A., Alharbi S.A., Koh A.P.F. (2018). Thymoquinone Inhibits Bone Metastasis of Breast Cancer Cells Through Abrogation of the CXCR4 Signaling Axis. Front. Pharm..

[182] Furusato B., Mohamed A., Uhlén M., Rhim J.S. (2010). CXCR4 and Cancer. Pathol. Int..

[183] Zlotnik A. (2008). New Insights on the Role of CXCR4 in Cancer Metastasis. J. Pathol..

[184] Vandercappellen J., Van Damme J., Struyf S. (2008). The Role of CXC Chemokines and Their Receptors in Cancer. Cancer Lett..

[185] Don-Salu-Hewage A.S., Chan S.Y., McAndrews K.M., Chetram M.A., Dawson M.R., Bethea D.A., Hinton C.V. (2013). Cysteine (C)-X-C Receptor 4 Undergoes Transportin 1-Dependent Nuclear Localization and Remains Functional at the Nucleus of Metastatic Prostate Cancer Cells. PLoS ONE.

[186] Chen Q., Zhong T. (2015). The Association of CXCR4 Expression with Clinicopathological Significance and Potential Drug Target in Prostate Cancer: A Meta-Analysis and Literature Review. Drug Des. Dev. Ther..

[187] He C., Lu X., Yang F., Qin L., Guo Z., Sun Y., Wu J. (2019). LncRNA UCA1 Acts as a Sponge of MiR-204 to up-Regulate CXCR4 Expression and Promote Prostate Cancer Progression. Biosci. Rep..

[188] Zheng Z., Qiu K., Huang W. (2021). Long Non-Coding RNA (LncRNA) RAMS11 Promotes Metastatis and Cell Growth of Prostate Cancer by CBX4 Complex Binding to Top2alpha. Cancer Manag. Res..

[189] Shi X., Zhang W., Nian X., Lu X., Li Y., Liu F., Wang F., He B., Zhao L., Zhu Y. (2020). The Previously Uncharacterized LncRNA APP Promotes Prostate Cancer Progression by Acting as a Competing Endogenous RNA. Int. J. Cancer.

[190] Xu S., Yi X.-M., Tang C.-P., Ge J.-P., Zhang Z.-Y., Zhou W.-Q. (2016). Long Non-Coding RNA ATB Promotes Growth and Epithelial-Mesenchymal Transition and Predicts Poor Prognosis in Human Prostate Carcinoma. Oncol. Rep..

[191] Hu W., Wang Y., Fang Z., He W., Li S. (2021). Integrated Characterization of LncRNA-Immune Interactions in Prostate Cancer. Front. Cell Dev. Biol..

[192] Dong L., Ding H., Li Y., Xue D., Liu Y. (2018). LncRNA TINCR Is Associated with Clinical Progression and Serves as Tumor Suppressive Role in Prostate Cancer. Cancer Manag. Res..

[193] Ho S.-Y., Wu W.-S., Lin L.-C., Wu Y.-H., Chiu H.-W., Yeh Y.-L., Huang B.-M., Wang Y.-J. (2019). Cordycepin Enhances Radiosensitivity in Oral Squamous Carcinoma Cells by Inducing Autophagy and Apoptosis Through Cell Cycle Arrest. Int. J. Mol. Sci..

[194] Zhang X., Zhang Y., Mao Y., Ma X. (2018). The LncRNA PCAT1 Is Correlated with Poor Prognosis and Promotes Cell Proliferation, Invasion, Migration and EMT in Osteosarcoma. OncoTargets Ther..

[195] Işın M., Uysaler E., Özgür E., Köseoğlu H., Şanlı Ö., Yücel Ö.B., Gezer U., Dalay N. (2015). Exosomal LncRNA-P21 Levels May Help to Distinguish Prostate Cancer from Benign Disease. Front. Genet..

[196] Kashyap D., Tuli H.S., Yerer M.B., Sharma A., Sak K., Srivastava S., Pandey A., Garg V.K., Sethi G., Bishayee A. (2021). Natural Product-Based Nanoformulations for Cancer Therapy: Opportunities and Challenges. Semin. Cancer Biol..

[197] Hussain Y., Mirzaei S., Ashrafizadeh M., Zarrabi A., Hushmandi K., Khan H., Daglia M. (2021). Quercetin and Its Nano-Scale Delivery Systems in Prostate Cancer Therapy: Paving the Way for Cancer Elimination and Reversing Chemoresistance. Cancers.

[198] Lu X., Chen D., Yang F., Xing N. (2020). Quercetin Inhibits Epithelial-to-Mesenchymal Transition (EMT) Process and Promotes Apoptosis in Prostate Cancer via Downregulating LncRNA MALAT1. Cancer Manag. Res..

[199] Termini D., Den Hartogh D.J., Jaglanian A., Tsiani E. (2020). Curcumin against Prostate Cancer: Current Evidence. Biomolecules.

[200] Liu T., Chi H., Chen J., Chen C., Huang Y., Xi H., Xue J., Si Y. (2017). Curcumin Suppresses Proliferation and In Vitro Invasion of Human Prostate Cancer Stem Cells by CeRNA Effect of MiR-145 and LncRNA-ROR. Gene.

[201] Mirzaei S., Paskeh M.D.A., Okina E., Gholami M.H., Hushmandi K., Hashemi M., Kalu A., Zarrabi A., Nabavi N., Rabiee N. (2022). Molecular Landscape of LncRNAs in Prostate Cancer: A Focus on Pathways and Therapeutic Targets for Intervention. J. Exp. Clin. Cancer Res..

[202] Prat J. (2012). Ovarian Carcinomas: Five Distinct Diseases with Different Origins, Genetic Alterations, and Clinicopathological Features. Virchows Arch..

[203] Baldwin L.A., Huang B., Miller R.W., Tucker T., Goodrich S.T., Podzielinski I., DeSimone C.P., Ueland F.R., van Nagell J.R., Seamon L.G. (2012). Ten-Year Relative Survival for Epithelial Ovarian Cancer. Obs. Gynecol..

[204] Zhang L., Luo M., Yang H., Zhu S., Cheng X., Qing C. (2019). Next-Generation Sequencing-Based Genomic Profiling Analysis Reveals Novel Mutations for Clinical Diagnosis in Chinese Primary Epithelial Ovarian Cancer Patients. J. Ovarian Res..

[205] Burges A., Schmalfeldt B. (2011). Ovarian Cancer: Diagnosis and Treatment. Dtsch. Arztebl. Int..

[206] Corrado G., Salutari V., Palluzzi E., Distefano M.G., Scambia G., Ferrandina G. (2017). Optimizing Treatment in Recurrent Epithelial Ovarian Cancer. Expert Rev. Anticancer Ther..

[207] Kwon M.J., Shin Y.K. (2013). Regulation of Ovarian Cancer Stem Cells or Tumor-Initiating Cells. Int. J. Mol. Sci..

[208] Meryet-Figuière M., Lambert B., Gauduchon P., Vigneron N., Brotin E., Poulain L., Denoyelle C. (2016). An Overview of Long Non-Coding RNAs in Ovarian Cancers. Oncotarget.

[209] Hu X., Feng Y., Zhang D., Zhao S.D., Hu Z., Greshock J., Zhang Y., Yang L., Zhong X., Wang L.-P. (2014). A Functional Genomic Approach Identifies FAL1 as an Oncogenic Long Noncoding RNA That Associates with BMI1 and Represses P21 Expression in Cancer. Cancer Cell.

[210] Zhao L., Ji G., Le X., Wang C., Xu L., Feng M., Zhang Y., Yang H., Xuan Y., Yang Y. (2017). Long Noncoding RNA LINC00092 Acts in Cancer-Associated Fibroblasts to Drive Glycolysis and Progression of Ovarian Cancer. Cancer Res..

[211] Zhang S., Leng T., Zhang Q., Zhao Q., Nie X., Yang L. (2018). Sanguinarine Inhibits Epithelial Ovarian Cancer Development via Regulating Long Non-Coding RNA CASC2-EIF4A3 Axis and/or Inhibiting NF-ΚB Signaling or PI3K/AKT/MTOR Pathway. Biomed. Pharm..

[212] Shang A., Wang W., Gu C., Chen C., Zeng B., Yang Y., Ji P., Sun J., Wu J., Lu W. (2019). Long Non-Coding RNA HOTTIP Enhances IL-6 Expression to Potentiate Immune Escape of Ovarian Cancer Cells by Upregulating the Expression of PD-L1 in Neutrophils. J. Exp. Clin. Cancer Res..

[213] Gordon M.A., Babbs B., Cochrane D.R., Bitler B.G., Richer J.K. (2019). The Long Non-Coding RNA MALAT1 Promotes Ovarian Cancer Progression by Regulating RBFOX2-Mediated Alternative Splicing. Mol. Carcinog..

[214] Liu S.-P., Yang J.-X., Cao D.-Y., Shen K. (2013). Identification of Differentially Expressed Long Non-Coding RNAs in Human Ovarian Cancer Cells with Different Metastatic Potentials. Cancer Biol. Med..

[215] Liu E., Liu Z., Zhou Y. (2015). Carboplatin-Docetaxel-Induced Activity against Ovarian Cancer Is Dependent on up-Regulated LncRNA PVT1. Int. J. Clin. Exp. Pathol..

[216] Worku T., Bhattarai D., Ayers D., Wang K., Wang C., Rehman Z.U., Talpur H.S., Yang L. (2017). Long Non-Coding RNAs: The New Horizon of Gene Regulation in Ovarian Cancer. Cell. Physiol. Biochem..

[217] Mani S.A., Guo W., Liao M.-J., Eaton E.N., Ayyanan A., Zhou A.Y., Brooks M., Reinhard F., Zhang C.C., Shipitsin M. (2008). The Epithelial-Mesenchymal Transition Generates Cells with Properties of Stem Cells. Cell.

[218] Hardin H., Zhang R., Helein H., Buehler D., Guo Z., Lloyd R.V. (2017). The Evolving Concept of Cancer Stem-like Cells in Thyroid Cancer and Other Solid Tumors. Lab. Investig..

[219] Chen S., Zhu J., Wang F., Guan Z., Ge Y., Yang X., Cai J. (2017). LncRNAs and Their Role in Cancer Stem Cells. Oncotarget.

[220] Adorno-Cruz V., Kibria G., Liu X., Doherty M., Junk D.J., Guan D., Hubert C., Venere M., Mulkearns-Hubert E., Sinyuk M. (2015). Cancer Stem Cells: Targeting the Roots of Cancer, Seeds of Metastasis, and Sources of Therapy Resistance. Cancer Res..

[221] Bregenzer M.E., Horst E.N., Mehta P., Novak C.M., Repetto T., Mehta G. (2019). The Role of Cancer Stem Cells and Mechanical Forces in Ovarian Cancer Metastasis. Cancers.

[222] Baccelli I., Trumpp A. (2012). The Evolving Concept of Cancer and Metastasis Stem Cells. J. Cell Biol..

[223] Buys S.S., Partridge E., Black A., Johnson C.C., Lamerato L., Isaacs C., Reding D.J., Greenlee R.T., Yokochi L.A., Kessel B. (2011). Effect of Screening on Ovarian Cancer Mortality: The Prostate, Lung, Colorectal and Ovarian (PLCO) Cancer Screening Randomized Controlled Trial. JAMA.

[224] Chang L., Ni J., Zhu Y., Pang B., Graham P., Zhang H., Li Y. (2019). Liquid Biopsy in Ovarian Cancer: Recent Advances in Circulating Extracellular Vesicle Detection for Early Diagnosis and Monitoring Progression. Theranostics.

[225] Redis R.S., Vela L.E., Lu W., Ferreira de Oliveira J., Ivan C., Rodriguez-Aguayo C., Adamoski D., Pasculli B., Taguchi A., Chen Y. (2016). Allele-Specific Reprogramming of Cancer Metabolism by the Long Non-Coding RNA CCAT2. Mol. Cell.

[226] Qiu J.-J., Lin Y.-Y., Ding J.-X., Feng W.-W., Jin H.-Y., Hua K.-Q. (2015). Long Non-Coding RNA ANRIL Predicts Poor Prognosis and Promotes Invasion/Metastasis in Serous Ovarian Cancer. Int. J. Oncol..

[227] Shi T., Gao G., Cao Y. (2016). Long Noncoding RNAs as Novel Biomarkers Have a Promising Future in Cancer Diagnostics. Dis. Mrk..

[228] Luo P., Liu X.-F., Wang Y.-C., Li N.-D., Liao S.-J., Yu M.-X., Liang C.-Z., Tu J.-C. (2017). Prognostic Value of Abnormally Expressed LncRNAs in Ovarian Carcinoma: A Systematic Review and Meta-Analysis. Oncotarget.

[229] Li J., Yu H., Xi M., Lu X. (2016). Long Noncoding RNA C17orf91 Is a Potential Prognostic Marker and Functions as an Oncogene in Ovarian Cancer. J. Ovarian Res..

[230] Li J., Huang H., Li Y., Li L., Hou W., You Z. (2016). Decreased Expression of Long Non-Coding RNA GAS5 Promotes Cell Proliferation, Migration and Invasion, and Indicates a Poor Prognosis in Ovarian Cancer. Oncol. Rep..

[231] Al-Rugeebah A., Alanazi M., Parine N.R. (2019). MEG3: An Oncogenic Long Non-Coding RNA in Different Cancers. Pathol. Oncol. Res..

[232] Tong J., Ma X., Yu H., Yang J. (2019). SNHG15: A Promising Cancer-Related Long Noncoding RNA. Cancer Manag. Res..

[233] Bhardwaj V., Tan Y.Q., Wu M.M., Ma L., Zhu T., Lobie P.E., Pandey V. (2021). Long Non-Coding RNAs in Recurrent Ovarian Cancer: Theranostic Perspectives. Cancer Lett..

[234] Bo H., Zhu F., Liu Z., Deng Q., Liu G., Li R., Zhu W., Tan Y., Liu G., Fan J. (2021). Integrated Analysis of High-Throughput Sequencing Data Reveals the Key Role of LINC00467 in the Invasion and Metastasis of Testicular Germ Cell Tumors. Cell Death Discov..

[235] Shanmugalingam T., Soultati A., Chowdhury S., Rudman S., Van Hemelrijck M. (2013). Global Incidence and Outcome of Testicular Cancer. Clin. Epidemiol..

[236] Albers P., Albrecht W., Algaba F., Bokemeyer C., Cohn-Cedermark G., Fizazi K., Horwich A., Laguna M.P., Nicolai N., Oldenburg J. (2015). Guidelines on Testicular Cancer: 2015 Update. Eur. Urol..

[237] Gashaw I., Dushaj O., Behr R., Biermann K., Brehm R., Rübben H., Grobholz R., Schmid K.W., Bergmann M., Winterhager E. (2007). Novel Germ Cell Markers Characterize Testicular Seminoma and Fetal Testis. Mol. Hum. Reprod..

[238] Houldsworth J., Reuter V., Bosl G.J., Chaganti R.S. (1997). Aberrant Expression of Cyclin D2 Is an Early Event in Human Male Germ Cell Tumorigenesis. Cell Growth Differ..

[239] Schmidt B.A., Rose A., Steinhoff C., Strohmeyer T., Hartmann M., Ackermann R. (2001). Up-Regulation of Cyclin-Dependent Kinase 4/Cyclin D2 Expression but down-Regulation of Cyclin-Dependent Kinase 2/Cyclin E in Testicular Germ Cell Tumors. Cancer Res..

[240] Statello L., Guo C.-J., Chen L.-L., Huarte M. (2021). Gene Regulation by Long Non-Coding RNAs and Its Biological Functions. Nat. Rev. Mol. Cell Biol..

[241] Bhan A., Soleimani M., Mandal S.S. (2017). Long Noncoding RNA and Cancer: A New Paradigm. Cancer Res..

[242] Yang N.-Q., Luo X.-J., Zhang J., Wang G.-M., Guo J.-M. (2016). Crosstalk between Meg3 and MiR-1297 Regulates Growth of Testicular Germ Cell Tumor through PTEN/PI3K/AKT Pathway. Am. J. Transl. Res..

[243] Guo J., Wang S., Jiang Z., Tang L., Liu Z., Cao J., Hu Z., Chen X., Luo Y., Bo H. (2022). Long Non-Coding RNA RFPL3S Functions as a Biomarker of Prognostic and Immunotherapeutic Prediction in Testicular Germ Cell Tumor. Front. Immunol..

[244] De Martino M., Chieffi P., Esposito F. (2021). MiRNAs and Biomarkers in Testicular Germ Cell Tumors: An Update. Int. J. Mol. Sci..

[245] Chovanec M., Lauritsen J., Bandak M., Oing C., Kier G.G., Kreiberg M., Rosenvilde J., Wagner T., Bokemeyer C., Daugaard G. (2021). Late Adverse Effects and Quality of Life in Survivors of Testicular Germ Cell Tumour. Nat. Rev. Urol..

[246] Cui Y., Miao C., Liu S., Tang J., Zhang J., Bu H., Wang Y., Liang C., Bao M., Hou C. (2021). Clusterin Suppresses Invasion and Metastasis of Testicular Seminoma by Upregulating COL15a1. Mol. Ther. Nucleic Acids.

[247] Andjilani M., Droz J.-P., Benahmed M., Tabone E. (2006). Down-Regulation of FAK and IAPs by Laminin during Cisplatin-Induced Apoptosis in Testicular Germ Cell Tumors. Int. J. Oncol..

[248] Dawson J.C., Serrels A., Stupack D.G., Schlaepfer D.D., Frame M.C. (2021). Targeting FAK in Anticancer Combination Therapies. Nat. Rev. Cancer.

[249] Li Q., Sun M., Wang M., Feng M., Yang F., Li L., Zhao J., Chang C., Dong H., Xie T. (2021). Dysregulation of Wnt/β-Catenin Signaling by Protein Kinases in Hepatocellular Carcinoma and Its Therapeutic Application. Cancer Sci..

[250] He Y., Sun M.M., Zhang G.G., Yang J., Chen K.S., Xu W.W., Li B. (2021). Targeting PI3K/Akt Signal Transduction for Cancer Therapy. Signal Transduct. Target. Ther..

[251] Tan S., Xu Y., Wang Z., Wang T., Du X., Song X., Guo X., Peng J., Zhang J., Liang Y. (2020). Tim-3 Hampers Tumor Surveillance of Liver-Resident and Conventional NK Cells by Disrupting PI3K Signaling. Cancer Res..

[252] Zhang X.-C., Wang J., Shao G.-G., Wang Q., Qu X., Wang B., Moy C., Fan Y., Albertyn Z., Huang X. (2019). Comprehensive Genomic and Immunological Characterization of Chinese Non-Small Cell Lung Cancer Patients. Nat. Commun..

[253] Stampouloglou E., Cheng N., Federico A., Slaby E., Monti S., Szeto G.L., Varelas X. (2020). Yap Suppresses T-Cell Function and Infiltration in the Tumor Microenvironment. PLoS Biol..

[254] Wang J., Huang F., Shi Y., Zhang Q., Xu S., Yao Y., Jiang R. (2021). RP11-323N12.5 Promotes the Malignancy and Immunosuppression of Human Gastric Cancer by Increasing YAP1 Transcription. Gastric Cancer.

[255] Zhu G., Ren D., Lei X., Shi R., Zhu S., Zhou N., Zu L., Mello R.A.D., Chen J., Xu S. (2021). Mutations Associated with No Durable Clinical Benefit to Immune Checkpoint Blockade in Non-S-Cell Lung Cancer. Cancers.

[256] Nachankar A., Krishnatry R., Joshi A., Noronha V., Agarwal J.P. (2013). Primary Mediastinal Seminoma; Resistance and Relapse: An Aggressive Entity. Indian J. Med. Paediatr. Oncol..

[257] Jacobsen C., Honecker F. (2015). Cisplatin Resistance in Germ Cell Tumours: Models and Mechanisms. Andrology.

[258] Wermann H., Stoop H., Gillis A.J.M., Honecker F., van Gurp R.J.H.L.M., Ammerpohl O., Richter J., Oosterhuis J.W., Bokemeyer C., Looijenga L.H.J. (2010). Global DNA Methylation in Fetal Human Germ Cells and Germ Cell Tumours: Association with Differentiation and Cisplatin Resistance. J. Pathol..

[259] Gan Y., Wang Y., Tan Z., Zhou J., Kitazawa R., Jiang X., Tang Y., Yang J. (2016). TDRG1 Regulates Chemosensitivity of Seminoma TCam-2 Cells to Cisplatin via PI3K/Akt/MTOR Signaling Pathway and Mitochondria-Mediated Apoptotic Pathway. Cancer Biol. Ther..

[260] Wei J., Gan Y., Peng D., Jiang X., Kitazawa R., Xiang Y., Dai Y., Tang Y., Yang J. (2018). Long Non-Coding RNA H19 Promotes TDRG1 Expression and Cisplatin Resistance by Sequestering MiRNA-106b-5p in Seminoma. Cancer Med..

[261] Polack F.P., Thomas S.J., Kitchin N., Absalon J., Gurtman A., Lockhart S., Perez J.L., Pérez Marc G., Moreira E.D., Zerbini C. (2020). Safety and Efficacy of the BNT162b2 MRNA Covid-19 Vaccine. N. Engl. J. Med..

[262] Han H.J., Nwagwu C., Anyim O., Ekweremadu C., Kim S. (2021). COVID-19 and Cancer: From Basic Mechanisms to Vaccine Development Using Nanotechnology. Int. Immunopharmacol..

